# PIKfyve activity is required for lysosomal trafficking of tau aggregates and tau seeding

**DOI:** 10.1016/j.jbc.2021.100636

**Published:** 2021-04-06

**Authors:** Alberto Carpinteiro Soares, Andreia Ferreira, Jonas Mariën, Charlotte Delay, Edward Lee, John Q. Trojanowski, Dieder Moechars, Wim Annaert, Louis De Muynck

**Affiliations:** 1 Neuroscience Department, Janssen Research and Development, a Division of Janssen Pharmaceutica NV, Beerse, Belgium; 2 VIB Center for Brain & Disease Research, Leuven, Belgium; 3 Department of Neurosciences, KU Leuven, Leuven, Belgium; 4 VIB Center for Medical Biotechnology, Ghent, Belgium; 5 Department of Biochemistry and Microbiology, Ghent University, Ghent, Belgium; 6 Department of Pathology and Laboratory Medicine, Institute on Aging and Center for Neurodegenerative Disease Research, University of Pennsylvania Perelman School of Medicine, Philadelphia, Pennsylvania, USA

**Keywords:** Alzheimer's disease, tauopathy, tau seeding, tau uptake, PIKfyve, Rac1, AD, Alzheimer's disease, AF488, Alexa Fluor 488, DN, dominant negative, HSPG, heparan sulfate proteoglycan, MVB, multivesicular body, PFF, preformed fibril, PHF, paired helical filament

## Abstract

Tauopathies, such as Alzheimer's disease (AD), are neurodegenerative disorders characterized by the deposition of hyperphosphorylated tau aggregates. Proteopathic tau seeds spread through the brain in a temporospatial pattern, indicative of transsynaptic propagation. It is hypothesized that reducing the uptake of tau seeds and subsequent induction of tau aggregation could be a potential approach for abrogating disease progression in AD. Here, we studied to what extent different endosomal routes play a role in the neuronal uptake of preformed tau seeds. Using pharmacological and genetic tools, we identified dynamin-1, actin, and Rac1 as key players. Furthermore, inhibition of PIKfyve, a protein downstream of Rac1, reduced both the trafficking of tau seeds into lysosomes and the induction of tau aggregation. Our work shows that tau aggregates are internalized by a specific endocytic mechanism and that their fate once internalized can be pharmacologically modulated to reduce tau seeding in neurons.

Tauopathies are a group of neurodegenerative disorders characterized by the deposition of hyperphosphorylated tau aggregates. Notably among tauopathies are disorders such as Alzheimer's disease (AD) where the deposition occurs in the form of paired helical filaments (PHFs) (1, 2). Studies have shown a correlation between the spatial distribution and load of PHFs and the clinical symptoms depicted by patients (3, 4). In AD patients, it is known that tau pathology develops in a typical spatiotemporal pattern, with PHFs first appearing in the locus coeruleus and spreading rostrally to the hippocampus, temporal lobe, and finally culminating in the primary sensory cortex (4, 5, 6, 7). The sequential progression of the pathology in areas of the brain that are anatomically connected by neuronal projections has led to the development of the transsynaptic propagation hypothesis (8). Supporting *in vitro* and *in vivo* studies show that tau aggregation can be induced in a neuron by adding pathological tau aggregates. Once internalized by neurons, tau aggregates get in contact with monomeric tau and act as a seed upon which the aggregate will grow (9, 10, 11, 12). Furthermore our lab has showed that synaptic contacts promote tau pathology propagation *in vitro* (13).

Recent efforts by several groups have shed light how tau pathology propagates in the brain and how tau seeds move from one neuron to another, although the precise molecular mechanisms remain only partially understood. We have previously shown that tau aggregates can be internalized *via* endocytosis by blocking the maturation of early endosomes with the consequent trapping of seeds and that dynamin inhibition reduces the propagation of tau seeds (14). Others have demonstrated that internalized tau aggregates colocalize with markers for bulk endocytosis and are transported to lysosomes in neurons (15). More recently, it was shown that tau internalization depends on dynamin and actin but not on clathrin in cell lines and neurons (16, 17, 18). Moreover, tau aggregates internalization appears to be mediated through interaction with heparan sulfate proteoglycans (HSPG) and LRP1 (17, 19, 20, 21).

Endosomal trafficking and sorting attract increasing attention in the AD field, with growing evidence showing that disruption in these mechanisms have an important impact in the development of AD pathology (22, 23). This is underscored by genome-wide association studies and exome sequencing that identified risk genes involved in endocytic transport regulation, including *BIN1*, *PICALM*, *CD2AP*, and *SORLA1* (24, 25). While the pathogenic mechanisms behind endosomal dysfunction can be diverse, recent work showed that compromised function of endolysosomal routes explains the escape of seeds into the cytoplasm (26), whereas retromer stabilization reduces tau pathology in animal models (27). This work highlights the need to get a better understanding of the molecular mechanisms responsible for the internalization and trafficking of tau seeds in neurons.

In this work we have used fluorophore labeled preformed fibrils (PFFs) of recombinant truncated tau (K18^P301L^) to study in detail the molecular mechanisms that are responsible for the internalization of seeds in mouse primary hippocampal neurons and how modulating these pathways could interfere with tau seeding. Using genetic and pharmacological approaches, we were able to confirm that aggregated tau seeds are internalized in a clathrin-independent, but dynamin-1-dependent mechanism as well as show that actin and Rac family small GTPase 1 (Rac1) play an important role in this mechanism. While exploring the potential link between Rac1 and actin polymerization, we identified the Rac1 downstream phosphoinositide kinase PIKfyve as a regulator of the subcellular distribution of tau seeds but not of their uptake. Pharmacological inhibition of PIKfyve proved to successfully reduce seeding by K18 PFFs and AD PHFs while reducing the delivery of aggregates into lysosomes.

## Results

### Neuronal tau uptake is a clathrin-independent, but dynamin- and actin-dependent process

To study the molecular mechanisms governing the internalization of tau aggregates, we developed an imaging assay that uses K18 PFFs labeled with either a pH-sensitive dye—pHrodo, for live-cell imaging – or a pH-insensitive dye—Alexa Fluor 488 (AF488), for immunocytochemistry. Whereas pHrodo labeling allows the study of uptake and delivery of tau aggregates into acidic intracellular compartments (28), the pH-insensitive dye AF488 grants a better understanding of uptake and subcellular distribution of seeds.

**Figure 1:**
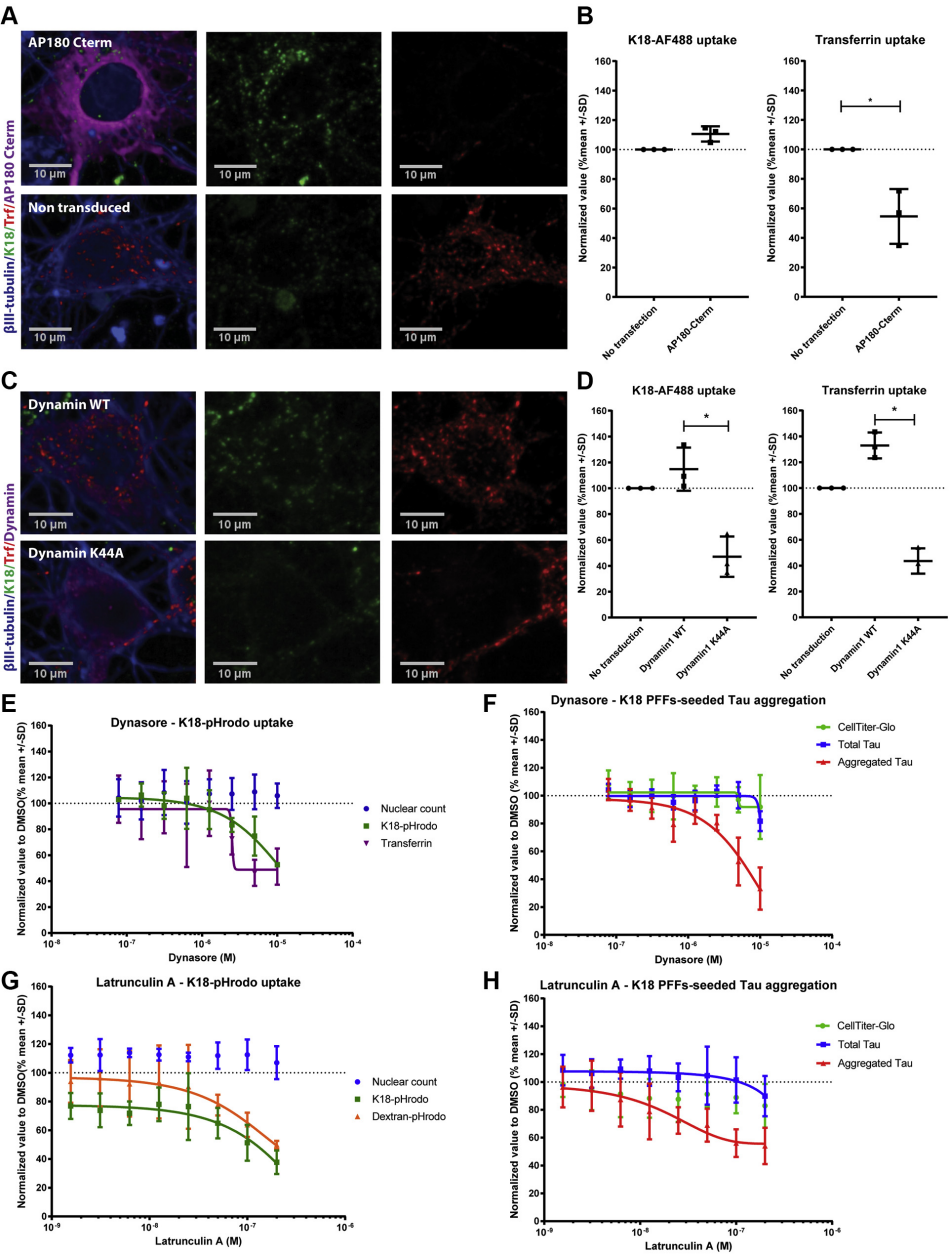
**Neuronal tau uptake is a clathrin-independent, but dynamin- and actin-dependent process.***A* and *B*, representative images of primary hippocampal neurons transfected with an AP180-Cterm construct and treated with 300 nM K18-AF488 and Transferrin-AF568 (Trf) for 30 min before fixation and staining, followed by puncta quantification (n = 3). *C* and *D*, representative images of primary hippocampal neurons transfected with Dyn1^WT^ or Dyn1^K44A^ mutant constructs and treated with 300 nM of K18-AF488 and Trf for 30 min before fixation followed by puncta quantification (one-way ANOVA, nonparametric test, ∗ <0.05, Kruskal–Wallis test, *p* = 0.0036, n = 3). *E*, imaging quantification of Trf and K18 pHrodo uptake in primary hippocampal cultures after 2-day pretreatment with dynasore in different concentrations (n = 3). *F*, CellTiter-Glo, total and aggregated tau measured in neurons treated with different concentrations of dynasore and 50 nM K18 PFFs to induce tau aggregation in neurons overexpressing tau:P301L (n = 3). *G*, quantification of dextran and K18 PFFs uptake in primary hippocampal cultures after 2-day pretreatment with Latrunculin A (n = 3). *H*, CellTiter-Glo, total and aggregated tau measured in neurons treated with Latrunculin A and 50 nM of K18 PFFs to induce tau aggregation in neurons overexpressing tau:P301L (n = 3).

Incubation of mouse primary hippocampal neurons with AF488 labeled K18 PFFs, followed by fixation at different incubation periods, shows that seeds are internalized within minutes of incubation and are quickly transported into late endosomal compartments including lysosomes (Fig. S1), similar to previous findings in human iPSC-derived neurons (18). Over time, K18 PFFs continue to be internalized by neurons, accumulating primarily in lysosomes and to a lesser extent in early endosomes. To extend our understanding of the endocytic routes of tau aggregates, we employed pharmacological and genetic tools to inhibit distinct and overlapping internalization routes in mouse primary neurons. Firstly, and to confirm the reported involvement of clathrin (16, 18), we overexpressed the C-terminal fragment of AP180, a high-affinity binder to clathrin, to outcompete clathrin's endogenous interactors, making the protein unavailable for its normal function (29). This resulted in a significant reduction of internalized transferrin (Trf), demonstrating that clathrin-mediated endocytosis was efficiently blocked. However, no significant reduction in uptake of K18 PFFs was observed (Fig. 1, *A* and *B*), indicating that the major entry route in neurons is also clathrin-independent, as shown in nonneuronal cells (16).

In contrast, overexpression of a dynamin-1-dominant negative mutant (*Dyn1*^K44A^) (30) in primary hippocampal neurons significantly reduced the uptake of K18 PFFs uptake as well as Trf internalization as compared with WT dynamin1 overexpression (Fig. 1, *C* and *D*). Likewise, pharmacological inhibition of dynamin-1 using dynasore (31) resulted not only in a reduced uptake of seeds but also diminished tau seeding in neurons at an IC_50_ of 3.6 μM in both assays (Fig. 1, *E* and *F*). These data further underscore that the uptake of K18 PFFs in primary hippocampal neurons is dynamin1-dependent as shown in iPSC-derived neurons (18).

Next, we investigated whether the uptake of K18 PFFs is actin-dependent by inhibiting actin polymerization using Latrunculin A (32). Treatment with Latrunculin A resulted in a dose-dependent reduction in dextran uptake, a bona fide marker for actin-dependent fluid-phase endocytosis. With respect to K18 PFFs uptake and tau aggregation, actin polymerization inhibition clearly resulted in a significant reduction of internalized K18 PFFs (IC_50_ = 57 nM) and consequently decreased significantly K18 PFFs-seeded tau aggregation (IC_50_ = 25 nM) (Fig. 1, *G* and *H*). These data contradict previous reports that claim that tau aggregates are internalized by an actin-independent mechanism in iPSC-derived neurons (18), but support data from other cell lines (16). Overall, these data give further support, but now in primary hippocampal neurons, that internalization of K18 PFFs is governed by a clathrin-independent, but dynamin1-and actin-dependent mechanism. Importantly, we now also link a reduced amount of tau seeds internalization to decreased tau seeding in neurons.

### Neuronal uptake of K18 PFFs requires actin polymerization through Rac1 activation

**Figure 2:**
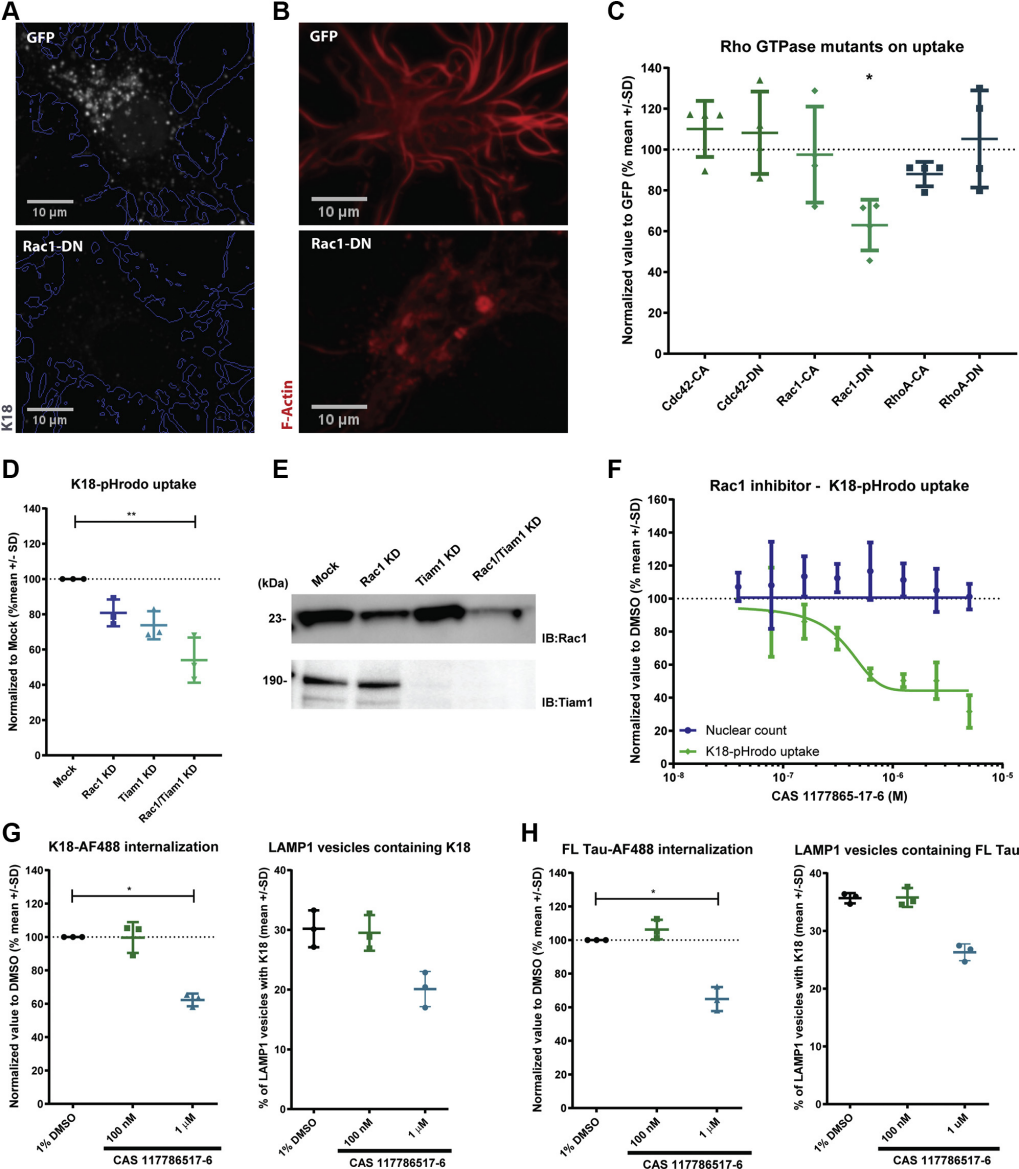
**Neuronal uptake of K18 PFFs requires actin polymerization through Rac1 activation.***A*, representative images of primary hippocampal neurons transfected with Rac1 dominant negative mutant or GFP as control and treated with 300 nM K18-AF488 for 16 h before fixation and imaging. *B*, representative images of primary hippocampal neurons cotransfected with Rac1 dominant negative mutant or GFP and LifeAct-RFP for F-actin visualization. *C*, quantification of K18-AF488 internalization in primary hippocampal cultures transfected with different GTPase mutants (one-way ANOVA, nonparametric test, ∗<0.05 Kruskal–Wallis test, *p* = 0.041, n = 3). *D*, imaging quantification of K18 PFFs uptake in hippocampal cultures transduced with LV-shRNAs against Rac1 and/or Tiam1 (one-way ANOVA, nonparametric test, ∗∗*p* < 0.05 Kruskal–Wallis test, *p* = 0.0006, n = 3). *E*, representative western blot analysis for Rac1 and Tiam1 in primary hippocampal cultures transduced with LV-shRNAs against Rac1 and/or Tiam1 for 7 days. *F*, imaging quantification of the uptake of K18-pHrodo in primary hippocampal cultures treated with the Rac1 inhibitor in dose–response (n = 3). Equal loading of cell lysates was evaluated with a total protein stain. Total protein staining (Cy5) full image can be found in Figure S3B. *G*, imaging quantification of K18-AF488 inside neurons and percentage of K18-AF488 inside LAMP1 vesicles on neurons treated with 1% DMSO, or 100 nM and 1 μM of YM-201636 (n = 3). *H*, imaging quantification of FL Tau-AF488 inside neurons and percentage of FL Tau-AF488 inside LAMP1 vesicles on neurons treated with 1% DMSO, or 100 nM and 1 μM of YM-201636 (n = 4).

Next, we aimed to identify additional downstream regulators of endocytosis to better understand the molecular mechanisms governing tau internalization. Actin has been demonstrated to be an important regulator of distinct forms of endocytosis depending on which regulator mediates its polymerization. Pivotal in this process is the family of Rho GTPases. Three proteins of this family have been functionally connected to endocytosis: RhoA, Cdc42, and Rac1 (33, 34). To investigate if any of these small GTPases is capable of modulating the internalization of tau seeds *via* actin polymerization, we overexpressed constitutively active (CA) or dominant negative (DN) mutants of all three proteins. At the same time, we assessed actin polymerization in neurons using LifeAct-RFP, a small 17-amino-acid-long actin marker that allows F-actin visualization (35). While all the tested mutants were able to change the pattern of LifeAct-RFP (Fig. S2), indicative of actin cytoskeleton modulation, only one mutant significantly reduced K18 PFFs internalization, namely Rac1-DN (Fig. 2, *A*–*C*). These data suggest that Rac1-dependent actin polymerization positively mediates internalization of K18 PFFs. Other modulators, although affecting the actin network architecture, did not interfere with the seed uptake mechanism. Next, we silenced the expression of Rac1 and Tiam1 by transducing primary hippocampal cultures with shRNAs using lentiviral vectors (LV-shRNA). Knockdown efficiency was confirmed by western blot and RT-qPCR (Figs. 2*E* and S3). Single knockdown of either Rac1 or Tiam1 reduced K18 PFFs internalization with 20% and 27%, respectively. Interestingly, when both proteins were silenced simultaneously, a cumulative 47% reduction in K18 PFFs uptake was observed (Figs. 2*D*). These data are likely a consequence of the fact that Rac1 is highly expressed in neurons, and even though we were able to reduce its mRNA expression by 90% (Fig. S3), the protein levels were only reduced by 50%. Thus, the remaining protein may still be able to modulate actin polymerization and endocytosis. Finally, we decided to study how Rac1 inhibition could affect Tau seeds uptake and distribution. We applied a pharmacologic approach using compound CAS 1177865-17-6, a molecule that reduces Rac1 activation by inhibiting the interaction with its activator Tiam1 (36). CAS 1177865-17-6 reduced K18-pHrodo signal in neurons in a dose-dependent manner (IC_50_ = 365 nM), confirming that K18 PFFs internalization is positively regulated by Rac1 activity (Fig. 2*F*). We also used the Rac1 inhibitor CAS 1177865-17-6 and quantified the amount of K18 and FL Tau-A488 inside neurons as well as the colocalization with LAMP1 (Fig. 2, *G* and *H*). Neurons were treated with concentrations of 100 nM and 1 μM since these were inactive and active concentrations in the pHrodo internalization assay respectively. As expected, 1 μM treatment with the Rac1 inhibitor reduces the internalization of both K18-AF488 and FL Tau-AF48, but not 100 nM, in line with the previous data (Fig. 2, *G* and *H*). Interestingly, Rac1 inhibition reduced the transport of K18 and FL Tau into lysosomes by 33% and 25% respectively, suggesting the involvement of this protein both in the uptake and the subcellular transport. Based on these data we conclude that Tiam1-dependent Rac1 activity plays a critical role in the uptake of K18 PFFs in primary hippocampal neurons.

**Figure 3:**
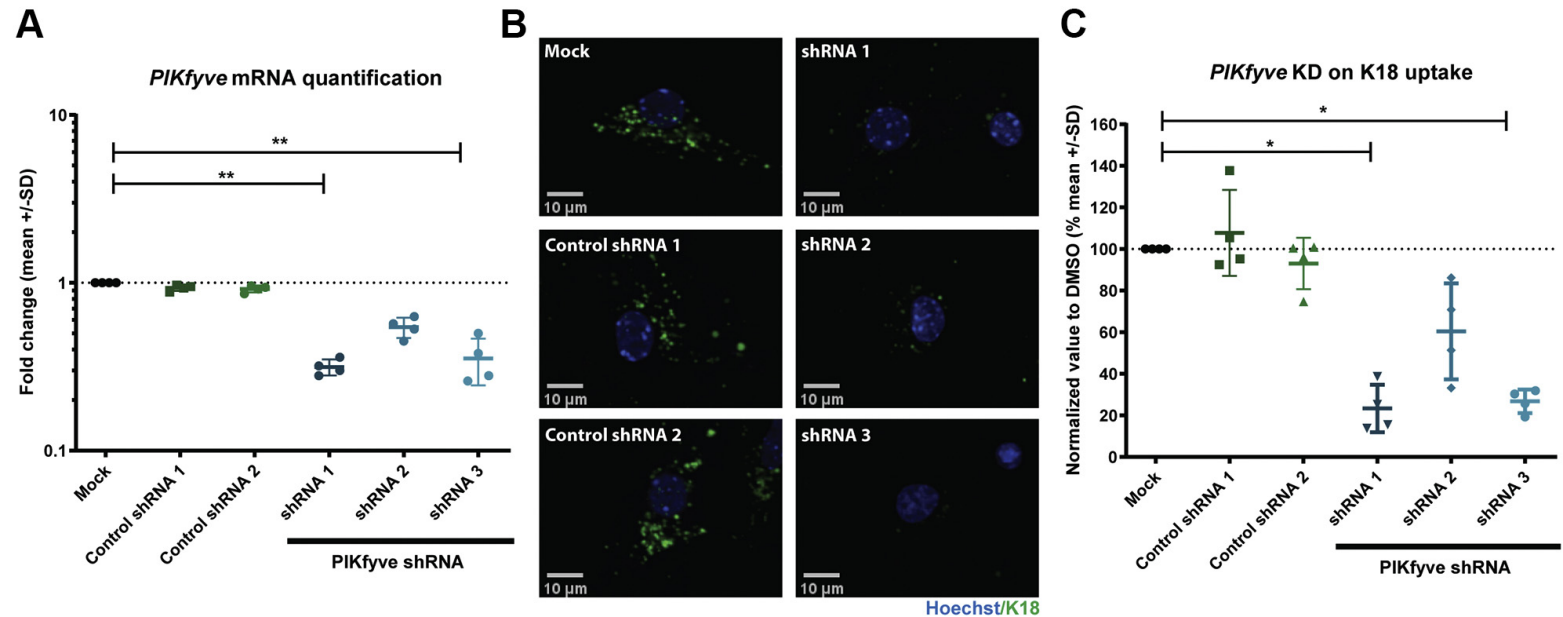
***Pikfyve* knockdown reduces K18 PFFs internalization to acidic vesicles.***A*, quantification of *Pikfyve* mRNA on sample treated with either control LV-shRNA or LV-shRNAs targeting *Pikfyve* (n = 4, mean fold change *versus* control). *B*, representative images of primary hippocampal cultures treated with either control LV-shRNAs or LV-shRNA against Pikfyve followed by incubation with K18-pHrodo and lysis for mRNA quantification. *C*, quantification of K18-pHrodo uptake in primary hippocampal cultures treated with different LV-shRNAs. (n = 4).

### *Pikfyve* knockdown reduces K18 PFFs internalization to acidic vesicles

We further explored the potential role of the downstream targets of Rac1 on the endocytosis of labeled K18 PFFs in primary neurons. Based on literature, we identified four downstream effectors that act on Rac1-dependent actin polymerization, namely Arp2/3, N-WASP, PAK1, and the PIP5K kinase family (37, 38, 39). We modulated the activity of these proteins using either pharmacological or genetic approaches. Arp2/3 and N-WASP act together in the branching of the actin cytoskeleton in neurons (40) and are known players in endosomal vesicle formation (41). However, pharmacological inhibition of Arp2/3 and N-WASP showed no effect on K18-pHrodo internalization in primary hippocampal cultures (Fig. S4, *A* and *B*). Likewise, overexpression of DN or constitutively active mutants of PAK1, a protein highly expressed in the brain that mediates actin polymerization by inhibiting cofilin, a known mediator of actin depolymerization (42), did not modulate K18 PFFs internalization in our assays (Fig. S4*C*).

Finally, we investigated the phosphoinositide kinase family, PIP5K (43). Within the PIP5K family, subfamilies can be distinguished, which include PIP5K (isoforms a,b,c) and PIP4K (isoforms a,b,c), which synthetize PI_4,5_P_2_ (44), and PIKfyve, responsible for PI_3,5_P_2_ formation (45). PI_4,5_P_2_ modulates the initial steps of endocytosis through different mechanisms, including the modulation of actin polymerization by sequestering of actin binders (46); recruitment of proteins that mediate membrane curvature (*e.g.*, BIN1) (47), and interaction with specific heparan sulfate proteoglycans (HSPG), glycan-coated proteins that are reported to interact with K18 PFFs (17). On the other hand, PI_3,5_P_2_ regulates endosomal vesicle maturation, in particular the maturing steps between the multivesicular body (MVB), late endosomes and lysosomes, including associated recycling events (48). We decided to target the different subfamilies of PIP5K using LV-shRNAs and test how these affect the process of K18 PFFs internalization using live-cell imaging. After imaging, samples were lysed and effective knockdown was confirmed through RT-qPCR. We tested the efficacy of three distinct LV-shRNAs in knocking down *Pikfyve*. LV-shRNA1 and 3 efficiently knocked down *Pikfyve* resulting in a concomitant significant decrease in K18-pHrodo signal in neurons. LV-shRNA2 was less effective in the knockdown of *Pikfyve*, giving rise to likewise less K18-pHrodo signal. (Fig. 3, *B* and *C*). Furthermore, none of the control LV-shRNA used could affect *Pikfyve* mRNA expression of K18-pHrodo signal. These data suggest that PIKfyve function may be an important regulator in K18 PFFs internalization. Data on the effect of other PIP5K family knockdown can be consulted in Figure S5. Because the PIKfyve loss of function elicited the highest reduction in the K18-pHrodo signal, we decided to continue our study focusing on PIKfyve.

### Pharmacological inhibition of PIKfyve reduces seeding by K18 PFFs and AD PHFs

To further study the potential role of PIKfyve in K18 PFFs uptake and seeding, we used YM-201636, a selective pharmacological PIKfyve inhibitor, shown to reduce the synthesis of PI_3,5_P_2_ and to a lower extent PI_4,5_P_2_ (49). To confirm its selectivity, we characterized this compound in a kinome profiling at 1 μM (Fig. S6). The selectivity profile confirmed strong binding to PIKfyve and no binding to the other two PIP5K subfamilies (note that the KINOMEscan from DiscoverX does not contain assays for the isoforms PIP5K1B and PIP4K2A—full data set is provided in Fig. S6).

**Figure 4:**
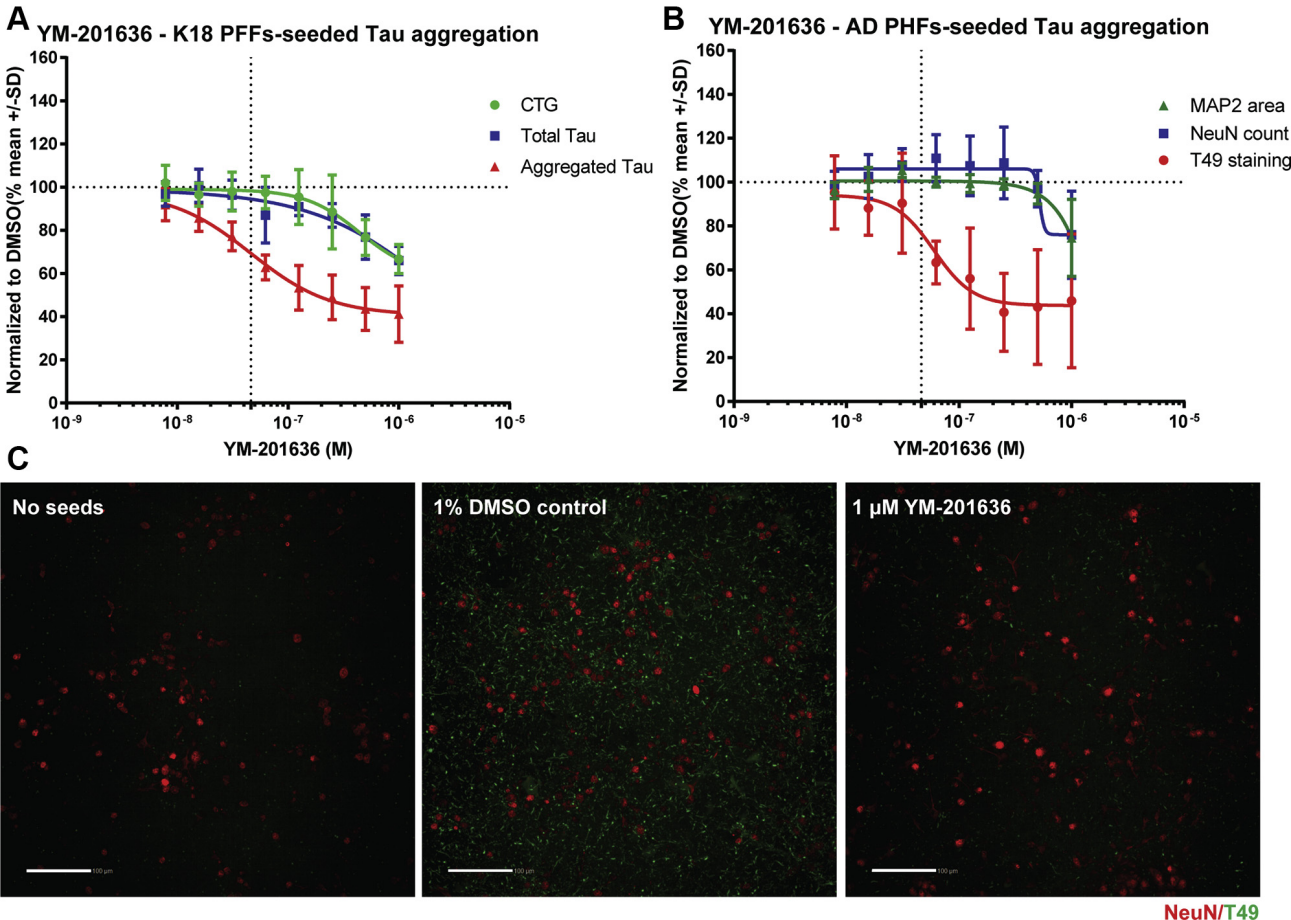
**Pharmacological inhibition of PIKfyve reduces seeding by K18 PFFs and AD PHFs.***A*, CellTiter-Glo, total and aggregated tau measured in neurons treated with different concentrations of YM-201636 and 50 nM of K18 PFFs to induce tau aggregation in neurons overexpressing tau:P301L (n = 4). *B*, T49, NeuN, and MAP2 area quantification of neurons treated with different concentrations of YM-201636 and AD PHFs (n = 3). *C*, representative images of primary hippocampal neurons treated with or without AD PHFs and 1% DMSO or YM-201636 (n = 3, scale bar: 50 μm).

We next tested if inhibition of PIKfyve by YM-201636 could reduce the seeding of tau aggregation in mouse primary neurons. After treatment with YM-201636, the load of seeded tau aggregates was significantly reduced following aggregation induction with K18 PFFs (Fig. 4*A*). The tau aggregation IC_50_ of 46 nM closely matches the IC_50_ for PIKfyve described in the literature of 33 nM (49). At concentrations higher than 500 nM, a moderate effect was noticed on cell viability and total tau, thereby indicating some cell toxicity. This can be explained by the long incubation time of the compound (9 days). To confirm that PIKfyve inhibition can result in a reduction of K18 PFFs-seeded tau aggregation, we used Apilimod, another potent, but structurally distinct, PIKfyve small-molecule inhibitor (50). Selective PIKfyve inhibition was confirmed in the kinome profile data. In our K18 PFFs-seeded tau aggregation assay, Apilimod also strongly reduced the seeded aggregation of tau in neurons (IC_50_=1 nM) (Fig. S7), confirming that PIKfyve activity is required for K18 PFFs-seeded tau aggregation in neurons.

We aimed to confirm the role of PIKfyve activity in seeded tau aggregation by testing PIKfyve pharmacological inhibition in an aggregation assay that uses a more pathophysiological relevant type of tau seed. Recently, it was demonstrated that aggregation of endogenous mouse tau can be induced using PHFs isolated from AD patients (51). Given the clinical relevance, we treated primary hippocampal neurons with AD patient-derived PHFs in the presence or absence of YM-201636. Seeded tau aggregation was measured by imaging using the mouse tau-specific antibody T49 after methanol fixation to remove all the soluble tau protein. Treatment with YM-201636 significantly reduced the amount of aggregated tau with an IC_50_ of 67 nM while showing minimal cell toxicity (Fig. 4, *B* and *C*).

### PIKfyve inhibition does not change the uptake of labeled K18 PFFs in neurons

**Figure 5:**
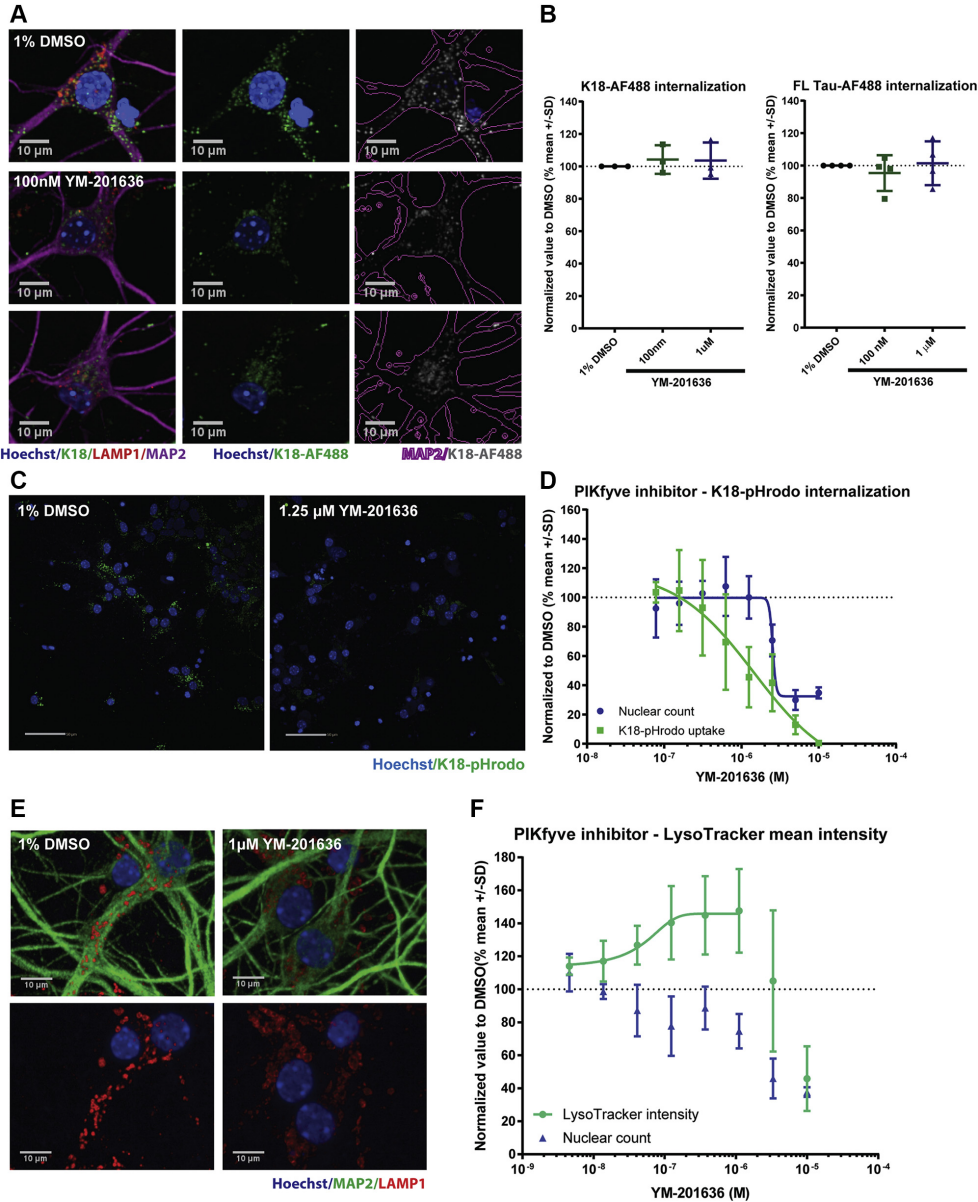
**PIKfyve inhibition does not change the uptake of labeled K18 PFFs in neurons.***A*, representative images of primary hippocampal neurons treated with 1% DMSO, 100 nM or 1 μM YM-201636 before 16 h incubation with K18-AF488 and fixation (n = 3). *B*, imaging quantification of the number of K18-AF488 and FL Tau-AF488 positive vesicles inside neurons treated with 1% DMSO, 100 nM or 1 μM YM-201636 (n = 3). *C*, representative images of primary hippocampal neurons treated with either 1% DMSO or YM-201636 before incubation and imaging of K18-pHrodo uptake (scale bar: 50 μm). *D*, imaging quantification of K18-pHrodo in primary hippocampal neurons treated with the YM-201636 inhibitor in dose–response (n = 4). *E*, representative images of primary hippocampal neurons treated either with 1% DMSO or YM-201636 before fixation and staining for LAMP1. *F*, image quantification of the LysoTracker positive vesicles intensity after treatment with different concentrations of YM-201636 (n = 3).

Our data thus far clearly point to PIKfyve playing a role in the process of tau seeding in neurons. In order to explore this, we decided to further characterize K18 PFFs uptake and internalization following PIKfyve inhibition. Using K18 PFFs labeled with the pH-insensitive AF488, the number of K18 or FL Tau PFF-positive vesicles in primary hippocampal neurons was to our surprise not altered at 100 nM or 1 μM YM-201636 treatment (Fig. 5, *A* and *B*). These results seemed at first to be counterintuitive given our previous data using pHrodo labeled K18 PFFs that suggested reduced internalization. To address this conundrum, we tested if pharmacological inhibition of PIKfyve would reduce K18-pHrodo signal as we observed using the knockdown approach. Similar to *PIKfyve* knock-down, treatment of primary hippocampal neurons with YM-201636 displayed a dose-dependent reduction in K18-pHrodo signal (Fig. 5, *C* and *D*). Since the pHrodo signal is not only dependent on neuronal internalization of a labeled substrate but also on the transport into low-pH compartments, we evaluated intracellular acidification using LysoTracker. Interestingly, YM-201636 treatment did not show a decrease, but rather an increase in LysoTracker intensity at concentrations up to 1 μM before reducing due to toxicity (Fig. 5*F*). These data show that the reduction in K18-pHrodo signal is not due to impaired acidification but instead suggest that K18 PFFs are retained in nonacidic vesicles in the endosomal pathway. In support of this notion, PIKfyve inhibition has been linked to an intracellular vesicular phenotype consisting in enlarged endosomes, caused by the reduced maturation of MVB into lysosomes as well as lysosomal recycling (52, 53). Indeed, when we treated primary hippocampal neurons with 1 μM YM-201636, we noticed a decrease in the number of LAMP1-positive organelles but an increase in their size, supported by 3D imaging and in line with previous reports (54, 55) (Figs. 5*E* and S8). Similar findings can be observed in HEK293T cells following 30 min treatment with 1 μM YM-201636, pointing to a cell-type-independent phenotype (Fig. S9). These data suggest that the reduction in K18 PFFs internalization previously seen with Rac1 inhibition is carried out by a mechanism that is PIKfyve-independent, while PIKfyve could be important for the fate of the seed once internalized.

### PIKfyve inhibition impairs the transport of K18 PFFs into lysosomes

**Figure 6:**
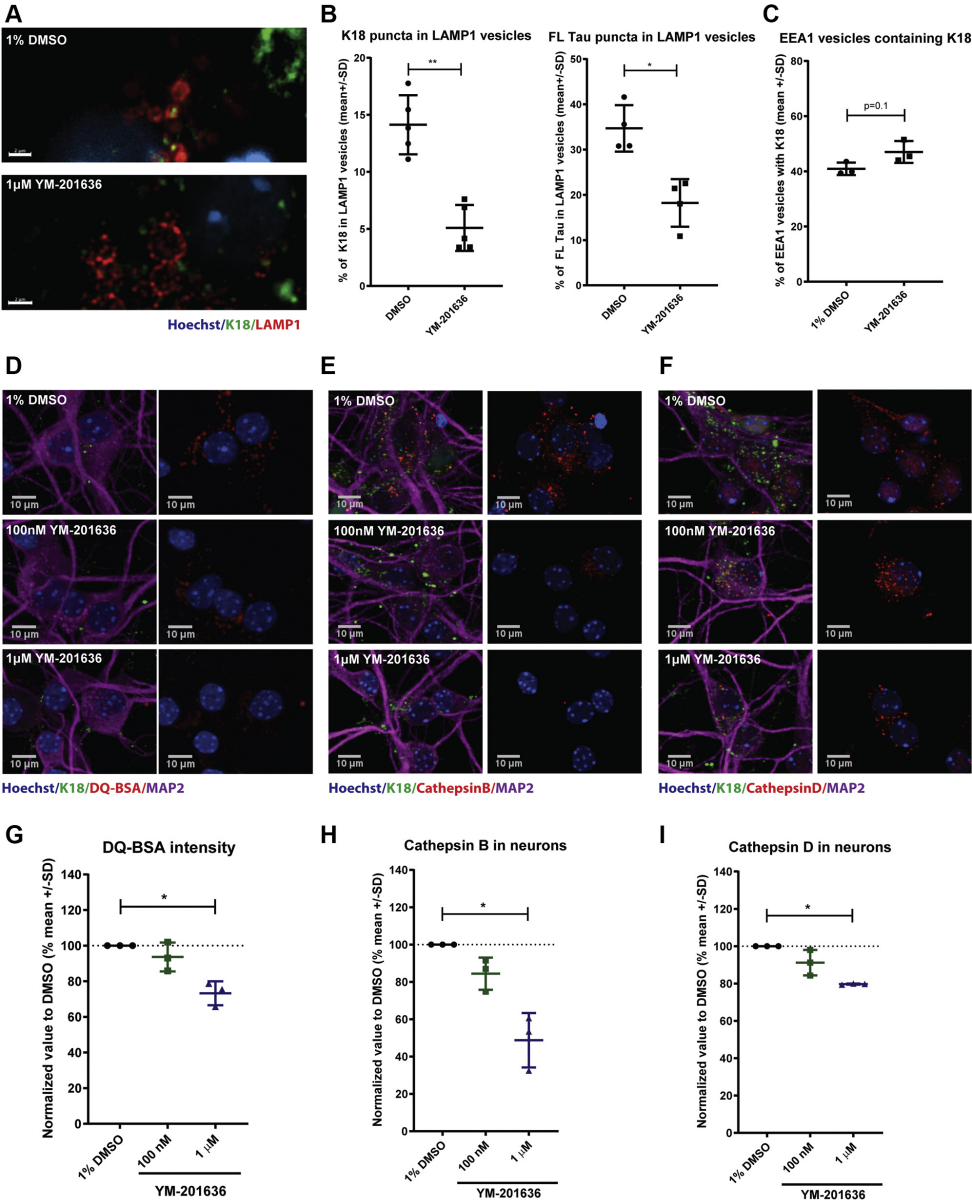
**PIKfyve inhibition impairs the transport of K18 PFFs into lysosomes**. *A*, representative images of primary hippocampal neurons treated with 1% DMSO or 1 μM YM-201636 before 16 h incubation with K18-AF488 and fixation and staining for LAMP1 (scale bar: 2 μm). *B*, image quantification of primary hippocampal neurons treated with 1% DMSO or 1 μM YM-201636 before a 16 h treatment with K18-AF488 or FL Tau-AF488 and fixation and staining for LAMP1 (Mann–Whitney test, ∗∗*p* < 0.05, n = 5 for LAMP1). *C*, image quantification of primary hippocampal neurons treated with 1% DMSO or 1 μM YM-201636 before 16 h treatment with K18-AF488 and fixation and staining for EEA1. *D*, representative images of mouse primary cultures treated with 1% DMSO, 100 nM or 1 μM YM-201636 before incubation with 20 μg/ml DQ-BSA (n = 3). *E* and *F*, representative images of mouse primary cultures treated with 1% DMSO, 100 nM or 1 μM YM-201636 and stained for cathepsin B and D respectively. *G*–*I*, imaging quantification of DQ-BSA puncta, cathepsin B and D inside neurons respectively (one-way ANOVA, nonparametric test, ∗<0.05, n = 3).

Since PIKfyve inhibition did not reduce the neuronal internalization of K18 PFFs, we hypothesized that the reduction in seeded tau aggregation might be caused either by a change in the fate of the seeds with consequent reduced release into the cytoplasm or by increased degradation. We started by evaluating how YM-201636 could affect the subcellular distribution of K18 PFFs in neurons. We treated primary hippocampal neurons with either 1% DMSO or 1 μM YM-201636 followed by incubation with AF488-labeled K18 PFFs for 16 h to allow internalization and transport into lysosomes. As mentioned before, LAMP1-positive organelles in neurons treated with YM-201636 appeared enlarged and reduced in number when compared with DMSO control (Fig. S8*B*). While there were no changes in the amount of K18 PFFs being internalized by neurons, the percentage of both K18 and FL Tau PFFs colocalizing with LAMP1 vesicles was reduced by 50% after YM-201636 treatment (Fig. 6, *A* and *B*). XYZ projection imaging shows that the majority of K18 PFFs can be seen close to LAMP1-positive vesicles, but not inside (Fig. S8*A*). On the other hand, K18 PFFs are slightly increased in number in EEA1-positive vesicles as well, thus pointing to a partial trapping or delayed transit of labeled K18 PFFs in earlier endosomal compartments. Because an absence of K18 PFFs in lysosomes could potentially be explained by an increased degradative capacity, we investigated lysosomal proteolytic activity. We used DQ-BSA, a self-quenchable substrate for hydrolase activity that, following endocytic uptake and delivery to degradative compartments, produces fluorescence upon cleavage. The intensity of DQ-BSA positive vesicles was decreased by 27% when primary hippocampal cultures were treated with 1 μM YM-201636, thus indicating reduced transport of cargo into proteolytic vesicles and consequently lowered degradation of substrates and excluding increased hydrolytic activity (Fig. 6*G*). YM-201636 treatment also reduced the number of cathepsin B- and D-positive vesicles, corroborating the DQ-BSA data and the idea of impaired degradation capacity in neurons (Fig. 6, *H* and *I*). Together, these data demonstrate that inhibition of PIKfyve prevents K18 PFFs to properly reach lysosomes. The observed reduction in K18 PFFs-mediated seeding could be a consequence of K18 PPFs no longer being capable of damaging lysosomes, which in turn could lead to a decrease in their release into the cytoplasm and hence reduced seeding capacity.

## Discussion

Tau pathology spreading over synaptically connected areas of the brain is a common feature in tauopathies (56). Many mechanisms have been proposed to explain the transfer of tau aggregates between neurons, but to this day there is still no clear view on which molecular mechanisms are involved in this process (57). Here we developed an imaging assay to study with high spatial and temporal resolution, the internalization and subsequent subcellular distribution of K18 PFFs in primary hippocampal neurons. Using biochemical and imaging-based Tau aggregation assays in the same cellular model allowed to prove the relevance of these mechanisms in the development of neuronal Tau aggregation.

Our work is consistent with that of others demonstrating that neuronal uptake of tau seeds occurs in a clathrin-independent, but dynamin1-and actin-dependent mechanism. Herein, we demonstrate for the first time the critical involvement of Rac1 in this process. The use of pharmacological and genetic approaches in neuronal uptake and seeding assays shows the relevance of this pathway in tau spreading pathology further strengthening findings by other groups. While we previously suggested CME in the neuronal uptake of Tau because of the demonstrated role of Bin1 (14), our current data prove that unequivocally clathrin does not play a role. Selectively inhibiting downstream effectors of Rac1 involved in actin polymerization and endocytosis, including Arp2/3, N-WASP, and PAK1, did not reduce tau seeds internalization. As mentioned before, clathrin-independent, dynamin-dependent endocytosis as well as macropinocytosis has been proposed previously as a mechanism by which tau aggregates are internalized by cell lines (16, 17) or human neurons (18). The data presented here show that also in neurons, several proteins that are part of the core machineries involved in clathrin-independent endocytosis or macropinocytosis, such as dynamin1, actin, and Rac1, are involved in the uptake of Tau seeds. However, molecular players functioning in macropinocytosis, like PAK1, could not be linked to tau seeds uptake (58, 59). Therefore, we conclude that the uptake of tau seeds by neurons is carried out by a distinct endocytosis mechanism that is dependent on dynamin and Rac1 and independent of clathrin.

Inhibition of the Rac1 downstream effector PIKfyve reduced seeding of both internalized K18 PFFs and AD PHFs with a very similar IC_50_ for the inhibitor YM-201636—46 nM *versus* 67 nM, respectively, and close to the biochemical IC_50_ of 33 nM for this compound (49). This demonstrates that internalized “synthetic” fibrils and pathophysiological AD seeds utilize the same route of transport once internalized in neurons, underscoring the relevance of this pathway in the context of Tauopathies.

When analyzing K18 PFFs internalization following PIKfyve inhibition, we observed pHrodo labeled K18 PFFs puncta were reduced in number and intensity. Because this was not observed when K18 PFFs were labeled with the pH-insensitive dye AF488, we hypothesized that the Rac1 effector PIKfyve likely exerts its role downstream of K18 PFFs neuronal uptake, *i.e.*, in routing internalized seeds toward lysosomes. The same results were obtained using FL Tau, thus giving strength to our hypothesis. PIKfyve is reported to be a critical regulator in the homeostasis of the endolysosomal system, among others in the reformation of lysosomes from endolysosomes, as is evidenced by the dramatic vacuolization of endosomal compartments when PIKfyve activity is inhibited (53, 60, 61). Our data show that in these circumstances, K18 PFFs stop being transported toward existing lysosomes and are most likely being entrapped in endosomes before being sorted to lysosomes. Endosomes entrapping K18 PFFs appear not to be acidified (explaining the apparent decreased uptake of pHrodo- but not AF488-K18 PFFs) nor LAMP1-positive compartments, although their nature remains to be further investigated.

Previous reports have consistently shown that tau seeds can be transported into lysosomes after neuronal internalization (15, 18, 62) and some linked lysosomal dysfunction to Tau aggregation (26, 63). Our lab has previously shown that internalized tau aggregates can result in the subsequent rupture of unidentified vesicles in neurons (14), enabling tau seeds to promote tau aggregation in the cytosol. Now, our present work identified the lysosomal delivery of Tau seeds as a key step in the process of seeded Tau aggregation. Based on our data we hypothesize that by reducing the transport of tau seeds into lysosomes *via* PIKfyve inhibition, tau seeding can be halted. In line with this idea it has been recently shown that exosomes containing Tau seeds require transport into the lysosome as well as lysosomal rupture to promote Tau aggregation (64). Whereas tau-seeds-induced rupture of lysosomes has not been shown yet, other protein oligomers such as α-synuclein fibrils are known to induce rupture of the lysosomal compartment and induce seeding (65).

Our data links for the first time PIKfyve activity to the delivery of tau seeds into lysosomes and implicates it as a player in tau seeding, which highlights the role of lipid metabolism in disease (66). Loss of PI_3,5_P_2_ in mice has been linked to neurodegeneration (67), while more recently it was shown that APP can bind and regulate the function of PIKfyve and consequently the generation of PI_3,5_P_2_ (68, 69). Of interest, PIKfyve inhibition has been show to rescue neurodegeneration in ALS patient iPSC-derived motor neurons and a C9orf72 ALS/FTD mouse model (70, 71) implying that the beneficial action of PIKfyve inhibition might not be limited to Tau aggregates. Furthermore, the PIKfyve inhibitor Apilimod has been used in several phase I clinical trials for the treatment of Crohn's disease, non-Hodgkin lymphoma, and more recently in the prevention of COVID-19 (clinicaltrials.com, alternative names: STA-5326 and LAM-002). Besides MVB maturation to lysosomes, PIKfyve and PI_3,5_P_2_ are also important for transport into the trans-Golgi network and for retromer function (72, 73, 74). Retromer stabilization using small-molecule chaperones can indeed reduce tau pathology in mouse models and tau phosphorylation in iPSC-derived human neurons (27, 75), thereby strengthening the idea of modulation of endolysosomal function as a disease-modifying strategy.

In conclusion, our work shows that in primary neurons, tau aggregates internalization and subcellular distribution occur *via* a pathway that can be modulated both genetically and pharmacologically. The understanding of the molecular mechanisms responsible for these endosomal routing opens opportunities for future therapeutic interventions to attenuate the spreading of tau pathology.

## Experimental procedures

### Cell culture

Mouse primary hippocampal neurons (mPHN) were isolated from E17 to E18 C57Bl/6J (Janvier) (IMSR Cat# JAX_000664, RRID:IMSR_JAX:000664) embryos, dissociated, and plated in 96-well plates (Greiner, μClear, 655946) previously coated with poly-L-lysine (Sigma Aldrich, P1274) at 20,000 cells per well. Neurons were kept at 37 °C and 5% CO_2_ in B-27 and GlutaMax supplemented Neurobasal medium (Gibco, 10888022) (76, 77).

QBI-HEK 293A (RRID:CVCL_6910) cells were cultured in DMEM (Gibco, 11960-044) supplemented with 10% FBS and 1% PenStrep (Gibco, 15140163). In total, 5000 cells were plated per well in 96-well plates and kept at 37 °C and 5% CO_2_. Forty-eight hours after plating, cells were treated with 1 μM YM-201636 and incubated for 30 min before being fixed with 4% paraformaldehyde and stained for LAMP1 (Abcam, Cat# ab25245, RRID:AB_449893) and F-actin (Thermo Fisher, Cat# A22287, RRID:AB_2620155)

### Generation and labeling of K18 PFFs

Recombinant N-terminal myc-tagged K18^P301L^ (truncated tau protein corresponding to the longest isoform between residues Q244 and E372) was produced in *Escherichia coli*. Fibrils were generated by incubating 40 μM K18^P301L^ protein with 40 μM low-molecular-weight heparin and 2 mM DTT in 100 mM sodium acetate at 37 °C. After 10 days the solution was centrifuged at 100,000*g* for 1 h at 4 °C. The pellet was resuspended in phosphate-buffered saline (PBS).

For labeling of the fibrils, K18^P301L^ fibrils were incubated for 1 h with either the pH-sensitive dye pHrodo (ThermoFisher, P35369) or the pH-insensitive dye AlexaFluor 488 or 568 (Thermo Fisher, A20000 or A20103 respectively). Excess free label was removed using dialysis against PBS for 72 h using a Slide-A-Lyzer Dialysis Cassettes, 10K MWCO (ThermoFisher, 66380).

### Imaging

For the live-cell imaging uptake assay, cultures were incubated with 50 nM of K18-pHrodo and 10 min before imaging Hoechst 33342 was added (final dilution = 1/15,000). For the uptake assay postfixation, cultures were incubated with 250 nM K18-Alexa Fluor 488 for different amounts of time, followed by fixation with 4% PFA for 1 h at room temperature followed by the immunocytochemistry protocol.

For immunocytochemistry, cells were fixed with 4% PFA for 15 min and blocked with 5% goat serum (Sigma, G9023). Incubation with antibodies was done in TBS-0.3% Tween-20 + 5% goat serum. Washing steps were done with TBS-0.3% Tween-20. For staining the following antibodies were used:

**Table:** 

Target	Catalogue number	Manufacturer	RRID
LAMP1	ab25245	Abcam	AB_449893
MAP2	ab5392	Abcam	AB_2138153
T49	MABN827	Millipore	AB_2848143
EEA1	3288S	Cell Signalling Technology	AB_2096811
Cathepsin D	ab75852	Abcam	AB_1523267
Cathepsin B	ab214428	Abcam	AB_2848144

For the LysoTracker assay, LysoTracker Deep Red (ThermoFisher, L12492) was diluted and added to the final concentration of 50 nM along with Hoechst 33342 (Thermo Fisher, H3570, final dilution = 1/15,000). Live cells were imaged using a 40× water immersion objective at 37 °C and 5%CO_2_.

For the lysosomal hydrolase assay, mouse hippocampal primary cultures were first incubated with respective treatment or DMSO control. Eight hours later, DQ-BSA (Thermo Fisher, D12051) was incubated at 20 μg/ml for 16 h before fixation with 4% PFA and immunocytochemistry for MAP2.

Imaging was performed on an Opera Phenix (PerkinElmer) instrument equipped with a 40× water immersion objective and by imaging at least 13 fields per well and four z-stack planes per field. Quantifications were performed on maximum intensity projections using the Harmony software (PerkinElmer). All data was normalized for cell viability in which case either number of nuclei (Hoechst 33342 staining) or MAP2-positive area was used.

### Cell cultures transfection, transductions, and pharmacological treatments

In experiments where WT and mutant constructs were overexpressed, transfections were done at 7 days *in vitro* (DIV7) using a calcium phosphate protocol. The following constructs were used:

**Table:** 

Protein	Mutant	Origin
Dynamin-1 WT	Dyn1^WT^	W. Annaert lab
Dynamin-1 dominant negative	Dyn1^K44A^	W. Annaert lab
AP180 dominant negative	AP180-CTerm	W. Annaert lab
Rac1 dominant negative	Rac1^N17^	W. Annaert lab
Rac1 constitutively active	Rac1^V12^	W. Annaert lab
Cdc42 WT	Cdc42^WT^	W. Annaert lab
Cdc42 dominant negative	Cdc42^N17^	W. Annaert lab
Cdc42 constitutively active	Cdc42^V12^	W. Annaert lab
RhoA dominant negative	RhoA^N19^	W. Annaert lab
RhoA constitutively active	RhoA^V14^	W. Annaert lab
Pak1 WT	Pak1	addgene (#12209, RRID:Addgene_12209)
Pak1 dominant negative	Pak1^H83LH86LK299R^	addgene (#12212, RRID:Addgene_12212)
Pak1 constitutively active	Pak1^L107F^	addgene (#26592, RRID:Addgene_26592)

Transductions with lentiviral vectors expressing different shRNAs were done at DIV6 and imaged at DIV14 to allow efficient knockdown. The following shRNAs (Sigma) were used:

**Table:** 

Target	TRCN	Targeted sequence
*Rac1*	TRCN0000055191	CGCAGACAGACGTGTTCTTAA
*Tiam1*	TRCN0000329080	CATTCGATCCTGCGAGATAAA
*Pip4k2a*	TRCN0000415033	TGGGAATGTACCGGCTTAATG
*Pip4k2b*	TRCN0000196947	GCAAGATCAAGGTGGACAATC
*Pip4k2c*	TRCN0000319782	GAGTCCTTCATCGATGTCTAT
*Pip5k1a*	TRCN0000271151	TCGTACTTTGCTGCCCAAATT
*Pip5k1b*	TRCN0000024585	CGGGCTATTACATGAATTTAA
*Pip5k1c*	TRCN0000378595	CGGCGAGAGCGACACATAATT
*Pikfyve* shRNA 1	TRCN0000150081	CGAGTTAAGGAGATCCTAATA
*Pikfyve* shRNA 2	TRCN0000196671	GTGACGATAATTTGGCTAATT
*Pikfyve* shRNA 3	TRCN0000146583	CGAACATTTACATGGGACAAA
Control shRNA 1	TRCN0000208001	∖
Control shRNA 2	TRCN0000072256	ACGCTGAGTACTTCGAAATGT

For the live-cell imaging, compounds were diluted in 100% DMSO and added on DIV6 followed by imaging at DIV9. The final volume of compound diluted in DMSO added to cells was 1% of the final volume. The following compounds were used:

**Table:** 

Compound name	CAS number	Target	Catalogue number	Manufacturer
Dynasore	1202867-00-2	Dynamin1		J&J internal
Latrunculin A	76343-93-6	Actin		J&J internal
NSC23766	1177865-17-6	Rac1	553502	Merck
YM-201636	371942-69-7	PIKfyve	ADVH3D686503	Sigma
Apilimod	541550-19-0	PIKfyve	ADVH0430B883	Sigma
CK-869	388592-44-7	Arp2/3		J&J internal
Wiskostatin	253449-04-6	N-WASP		J&J internal

### AlphaLISA immunoassay and CellTiter-Glo

For the neuronal aggregation assay using K18 PFFs and tau overexpression, mouse primary hippocampal cultures were transduced with AAV6-hSyn1-tau:P301L on DIV0, compounds were added on DIV1, and seeding was done at DIV3 using 50 nM of K18 PFFs. Following lysis in 80 μl RIPA buffer on DIV10, lysates were incubated for 2 h with the alphaLISA capture mixture: for aggregated tau, both streptavidin donor and acceptor beads were conjugated to the same tau antibodies; for total tau, the streptavidin donor and acceptor beads were conjugated to two different tau antibodies with close distinct epitopes.

For cell viability measures we quantified the amount of ATP in cell lysates using CellTiter-Glo (G9681, Promega). The manufacturer's protocol was followed, and luminescence was measured using an Envision plate reader (PerkinElmer).

### AD PHFs preparation and seeded tau aggregation assay

Human brain tissues from six histologically confirmed sporadic AD patients with abundant tau pathology (Braak staging V/VI) were provided by the Center for Neurodegenerative Disease Research brain bank at the University of Pennsylvania with informed consent from next of kin. The purification of the AD seeds from these brain sections was adapted from Guo *et al*. (51). In sum, 18 g of frontal cortical gray matter was homogenized using a Fastprep-24 Homogenizer and Lysing matrix D tubes (MP Biomedicals, 6953-050) and in nine volumes (v/w) of high-salt buffer (10 mM Tris-HCl, pH 7.4, 0.8 M NaCl, 1 mM EDTA), supplemented with 2 mM dithiothreitol [DTT], protease/phosphatase inhibitors (0.5 mM PMSF, 2.3 μM Leupeptin, 1.5 μM Pepstatin, 2.8 μM TPCK, 2.7 μM TLCK, 1 μg/ml Trypsin inhibitor from *Glycine max* (soybean), 0.1 μM EDTA, 2 mM imidazole, 1 mM activated sodium orthovanadate, 1 mM sodium fluoride), 0.1% sarkosyl, and 10% sucrose. The homogenates were centrifuged at 10,000*g* for 10 min at 4 °C. Pellets were re-extracted once using the same buffer conditions as the starting materials. The supernatants were pooled and filtered using a 40 μm cell strainer (Thermo Fisher, 08-771-1), and sarkosyl was added to the filtrate to reach 1%. After 1–1.5 h nutation at room temperature, samples were centrifuged at 150,000*g* for 75 min at 4 °C. The resulting 1% sarkosyl-insoluble pellets were washed once in PBS and then nutated overnight in PBS (90 μl/g Gy matter) at room temperature. The pellets were then resuspended by passing successively through 20, 23, and 26 G needles. The resuspended sarkosyl-insoluble pellets were further purified by sonication (A = 100%, C = 50%, Total Ws = 200) using a Vial tweeter Sonotrode (Hielscher) followed by centrifugation at 100,000*g* for 30 min at 4 °C. The pellets were resuspended in PBS (20 μl per g of gray matter) by passing successively through 20, 23, and 26 G needles and sonicated again with the same settings as before. This suspension was then spun at 10,000*g* for 30 min at 4 °C to remove large debris. Supernatants of all six extractions were pooled after confirmation of seeding activity in primary neurons. The final pooled supernatants were used in the study and referred to as “AD PHFs.”

In the neuronal aggregation assay using AD PHFs, neurons were treated with compounds at DIV7 and 0.25 μl of AD PHFs per well on DIV9. Neurons were kept until DIV23 after which cells were fixed with methanol and stained with a monoclonal anti-tau antibody (Millipore, MABN827, RRID:AB_2848143).

### SDS-PAGE

Western blot was performed by running samples on 4–12% Bis-Tris gels (Bio-Rad, 3450124), and the primary antibodies were diluted in PBS plus 0.3% Tween-20 and 5% blotting grade blocker (Bio-Rad, 1706404). Membranes were incubated with antibodies against Rac1 (Millipore, 05-389, RRID:AB_309712) or Tiam1 (Novus, NB100-2301, RRID:AB_2271614). Total protein staining was performed by incubating samples with Amersham QuickStain (Amersham, RPN4000) using the manufacture protocol.

### RT-qPCR

For mRNA quantification, primary hippocampal neurons were lysed using RLT buffer. RNA was extracted using a RNeasy 96 kit (Qiagen, 74181) and following the manufacturer's protocol. Isolated RNA was converted to cDNA using the high-capacity cDNA reverse transcription kit (ThermoFisher, 4368814). RT-qPCR was performed in a QuantStudio 12K Flex (Applied Biosystems). Data was analyzed using the qbase+ software (Biogazelle, RRID:SCR_003370). The following TaqMan assays (IDT DNA) were used:

**Table:** 

Gene symbol	IDT DNA assay ID	RefSeq IDs targeted
*Rac1*	Mm.PT.58.32665455	NM_009007(1)
*Tiam1*	Mm.PT.58.42627491	NM_001145887(3)
*Pip5k1a*	Mm.PT.56a.8266696	NM_008847(1)
*Pip5k1b*	Mm.PT.58.33575655	NM_008846(1)
*Pip5k1c*	Mm.PT.56a.10269471	NM_001146687(2)
*Pip4k2a*	Mm.PT.56a.32326669	NM_008845(1)
*Pip4k2b*	Mm.PT.56a.6102090	NM_054051(1)
*Pip4k2c*	Mm.PT.58.46080863.g	NM_054097(1)
*Pikfyve*	Mm.PT.56a.6919895	NM_011086(1)
*Tbp*	Mm.PT.39a.22214839	NM_013684
*Gusb*	Mm.PT.39a.22214848	NM_010368
*Polr2a*	Mm.PT.39a.22214849	NM_009089
*Rplp0*	Mm.PT.58.43894205	NM_007475

### Statistical analysis

All data was analyzed using GraphPad Prism v7.0 (San Diego, www.graphpad.com, RRID:SCR_002798). Normal distribution was evaluated using the Shapiro–Wilk test. For comparisons between two groups, Mann–Whitney U test was used for non-Gaussian distribution samples. For multiple groups, Kruskal–Wallis test was used for non-Gaussian samples, followed by Dunn's multiple comparison post hoc test to compare between groups. Dose–response was analyzed using a nonlinear regression with a 95% confidence interval. *p*-Values were calculated with a 95% confidence internal, when nothing is mentioned there is no statistical significance, otherwise: ∗*p* < 0.05, ∗∗*p* < 0.005. Data presented was shown as mean ± SD.

## Data availability

All data present are available in the article and in the supporting information.

## Supporting information

This article contains supporting information.

## Conflict of interest

The authors declare that they have no conflicts of interest with the contents of this article.
