# Supplementary material for: PIKfyve activity is required for lysosomal trafficking of tau aggregates and tau seeding
Source: J Biol Chem. 2021 Apr 6;296:100636. doi: 10.1016/j.jbc.2021.100636 (PMC8134070; doi:10.1016/j.jbc.2021.100636)
Supplement: Figures S1 to S9 [file mmc1.pdf]

Supporting information

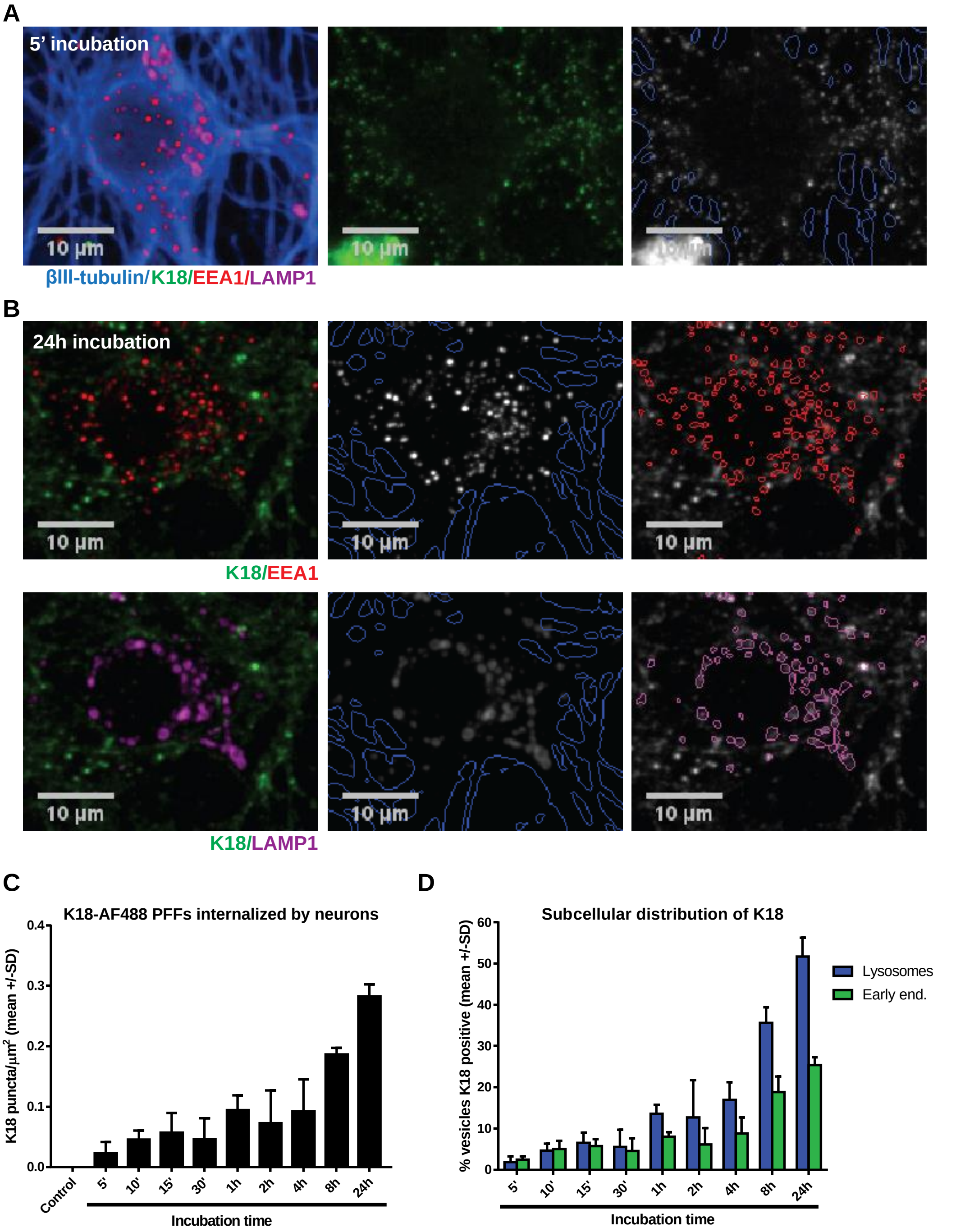

Supporting figure 1 – (A) Representative images of primary hippocampal cultures treated with 300nM K18-AF488 for five minutes before fixation and staining – right image represents K18 puncta inside masking (blue line) for βIII-tubulin. (B) Representative images of primary hippocampal cultures treated with 300nM K18-AF488 for 24 hours before fixation and staining. Upper row, middle image represents immunostaining for EEA1 to identify early endosomes inside neurons (blue line). Upper row, right image represents K18-AF488 puncta (white) inside EEA1-positive vesicles (red lines). Bottom row, middle image represents identification of LAMP1-positive vesicles inside neurons (blue line). Bottom row, right image represents K18-AF488 punctae (white) inside LAMP1-positive vesicles (purple lines). (C) Quantification of K18-AF488 puncta inside of neurons after different incubation times. (D) Quantification of the percentage of LAMP1- and EEA1-positive vesicles containing K18-AF488 puncta after different incubation times.

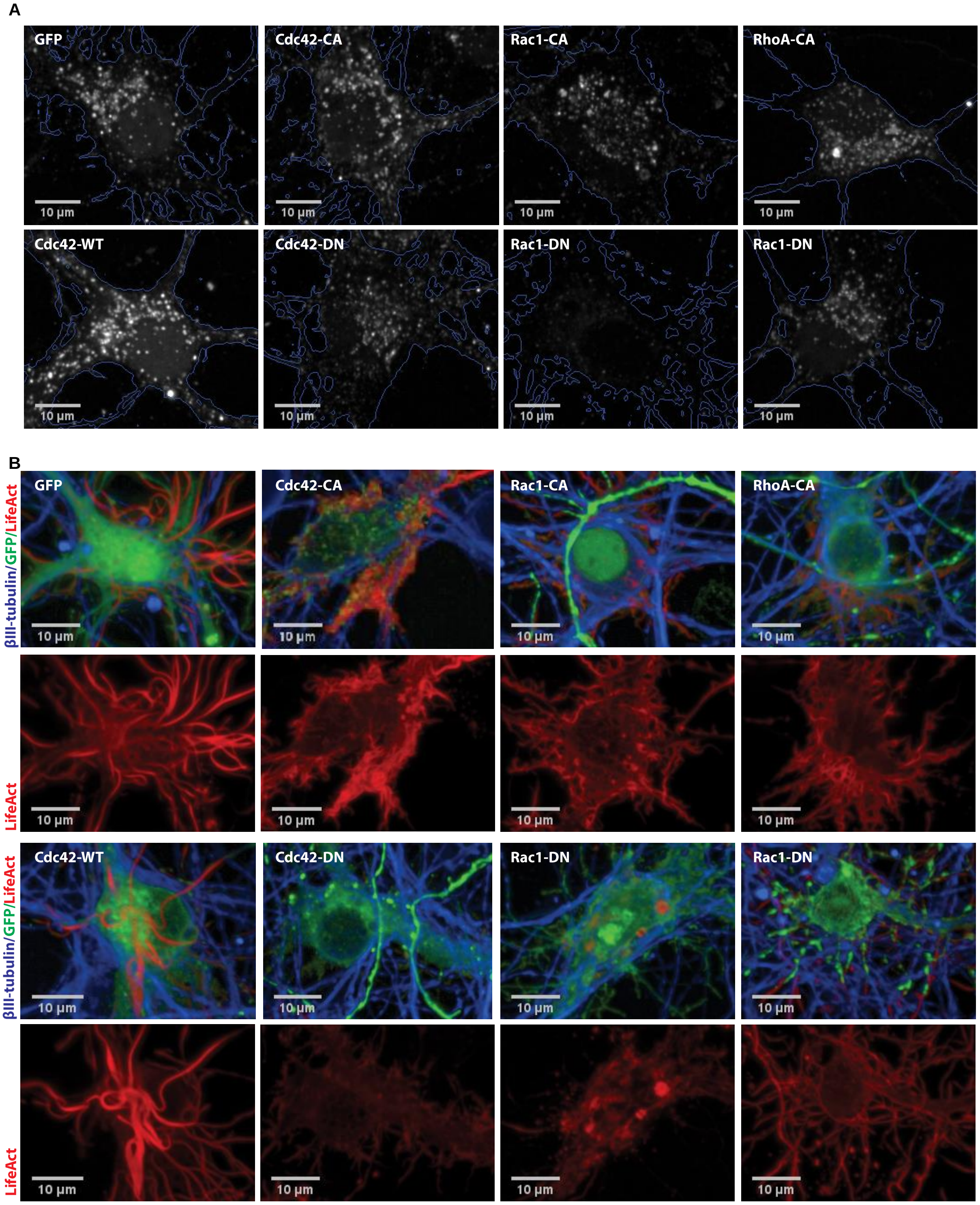

Supporting figure 2 – (A) Representative images of primary hippocampal cultures transfected with GFP-tagged constitutive active (CA) or dominant negative (DN) forms of RhoA, Rac1 and Cdc42 plus GFP as control. Cells were treated with 300nM K18-AF58 before fixation and immunostained for βIII-tubulin (blue line represents neuronal masking and white dots K18-AF568). (B) Representative images of primary hippocampal cultures co-transfected with GFP-tagged constitutive active (CA) or dominant negative (DN) forms of RhoA, Rac1 and Cdc42. GFP transfection is included as a control and co-transfection with LifeAct-RFP for F-actin visualization. The same representative images for GFP and Rac1-DN are present in figure 2.

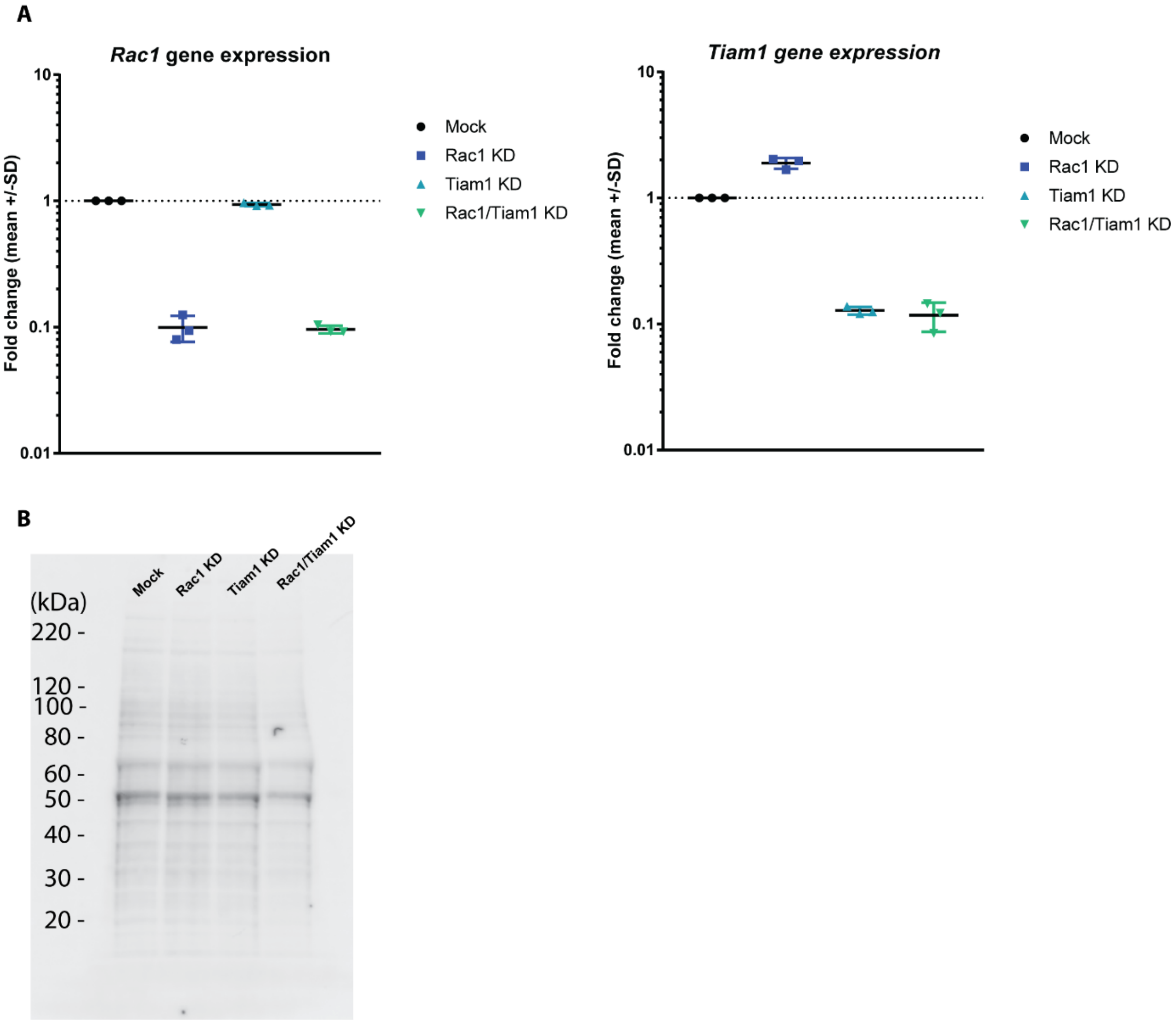

Supporting figure 3 – (A) *Rac1* and *Tiam1* mRNA expression quantification of primary hippocampal cultures treated with LV-shRNA (5MOI) against *Rac1*, *Tiam1* or both (n=3). (B) Representative total protein staining of Western blot in primary hippocampal cultures transduced with LV-shRNAs against Rac1 and/or Tiam1 for 7 days.

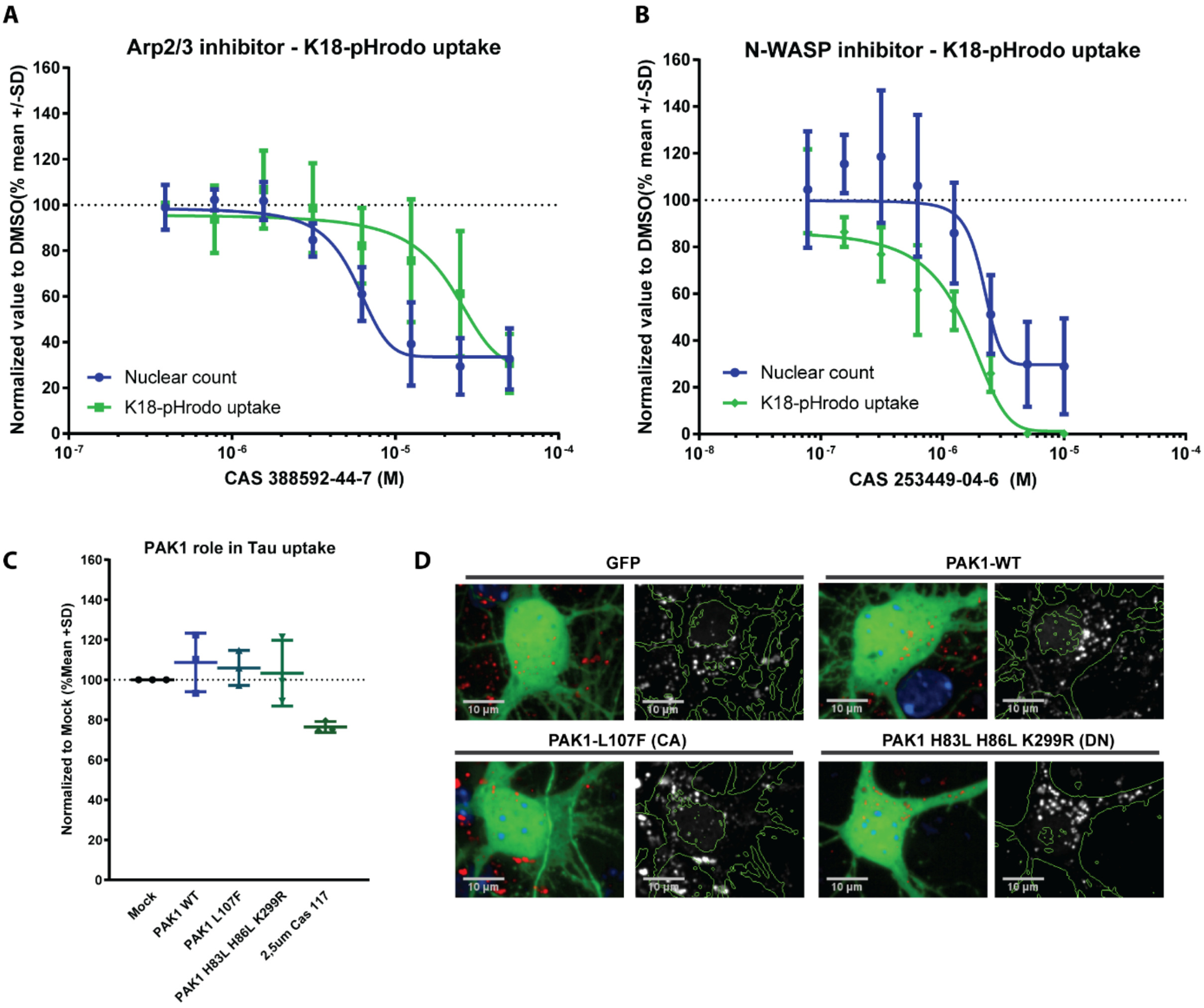

Supporting figure 4 – (A) Quantification of K18 PFFs uptake in primary hippocampal cultures treated with Arp2/3 inhibitor CAS 388592-44-7 (n=3). (B) Quantification of K18 PFFs uptake in primary hippocampal cultures treated with N-WASP inhibitor CAS 253449-04- (n=3). (C and D) Quantification and representative images of primary hippocampal cultures transfected with GFP or GFP-tagged PAK1 WT and mutant forms of PAK1. Neurons were treated with 300nM K18-AF568 before being fixed and stained (n=3).

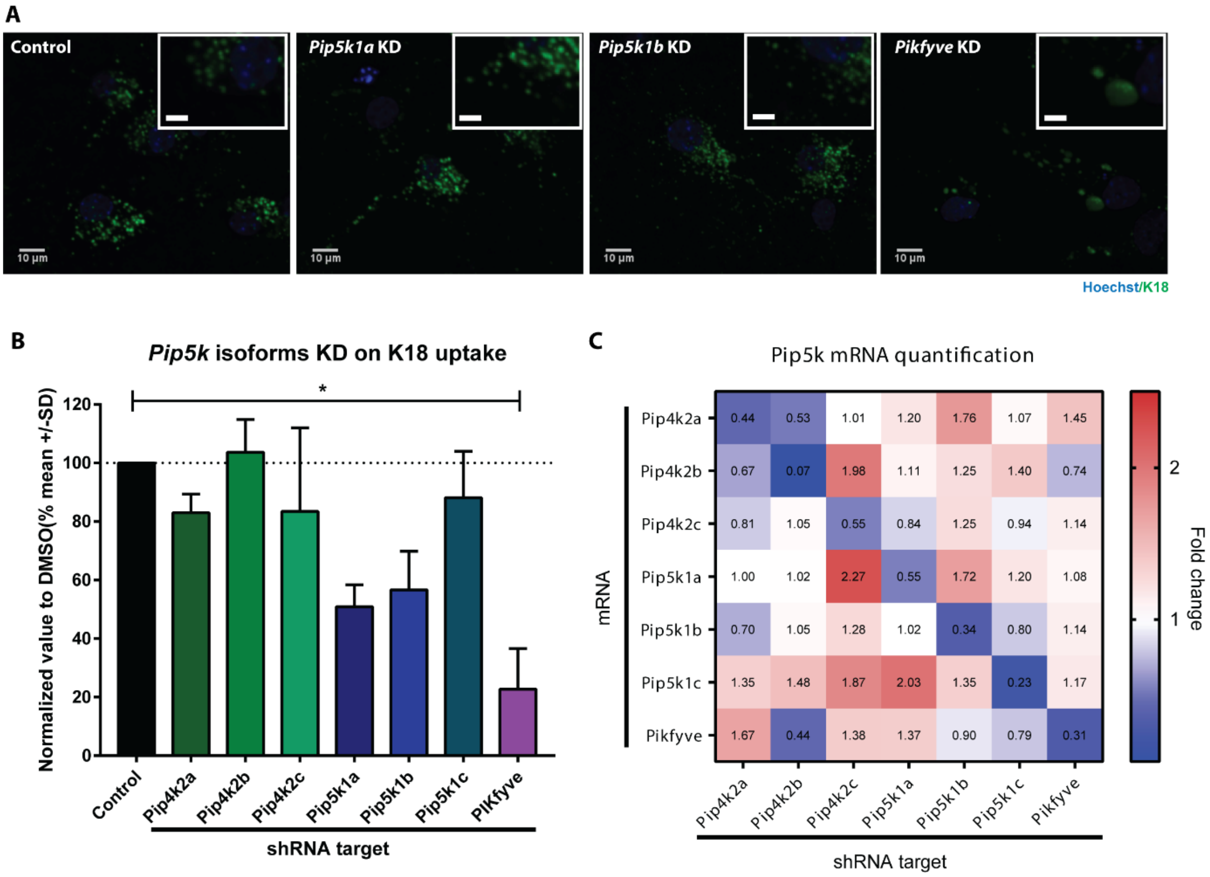

mRNA \ shRNA target	Pip4k2a	Pip4k2b	Pip4k2c	Pip5k1a	Pip5k1b	Pip5k1c	Pikfyve
Pip4k2a	0.44	0.53	1.01	1.20	1.76	1.07	1.45
Pip4k2b	0.67	0.07	1.98	1.11	1.25	1.40	0.74
Pip4k2c	0.81	1.05	0.55	0.84	1.25	0.94	1.14
Pip5k1a	1.00	1.02	2.27	0.55	1.72	1.20	1.08
Pip5k1b	0.70	1.05	1.28	1.02	0.34	0.80	1.14
Pip5k1c	1.35	1.48	1.87	2.03	1.35	0.23	1.17
Pikfyve	1.67	0.44	1.38	1.37	0.90	0.79	0.31

Supporting figure 5 – (A) Representative images of primary hippocampal neurons treated with a mock control or shRNA against the isoforms Pip5k1a, Pip5k1b and Pikfyve. Inserts represent an amplified view on vesicles containing K18-pHrodo (scale bar of inserts: 5 µm). (B) Imaging quantification of the number of K18 PFFs-positive vesicles inside of neurons after transduction with LV-shRNAs against the different isoforms of *Pip5k* and following treatment with 50nM K18-pHrodo. Data are normalized to no transfection control condition (n=3). (C) mRNA quantification of the different *Pik5k* isoforms in primary hippocampal neurons of cells treated with different shRNA against the different isoforms.

Supporting figure 6 - Kinome profile of compound YM-201636 at 1µM. The assay is a competition assay, 100% control refers to no binding to the proteins while 0% represents binding of the compound to all the protein available. (A) Table with data for every kinase tested. (B) tree diagram with visual representation of the kinases hit by YM-201636.

DiscoverX Gene Symbol	Percent control	DiscoverX Gene Symbol	Percent control	DiscoverX Gene Symbol	Percent control
AAK1	100	CDK11	100	EPHA6	89
ABL1(E255K)-phosphorylated	58	CDK2	100	EPHA7	100
ABL1(F317I)-nonphosphorylated	94	CDK3	100	EPHA8	100
ABL1(F317I)-phosphorylated	76	CDK4	97	EPHB1	100
ABL1(F317L)-nonphosphorylated	69	CDK4-cyclinD1	88	EPHB2	100
ABL1(F317L)-phosphorylated	93	CDK4-cyclinD3	89	EPHB3	95
ABL1(H396P)-nonphosphorylated	83	CDK5	100	EPHB4	100
ABL1(H396P)-phosphorylated	75	CDK7	92	EPHB6	93
ABL1(M351T)-phosphorylated	98	CDK8	100	ERBB2	87
ABL1(Q252H)-nonphosphorylated	82	CDK9	100	ERBB3	82
ABL1(Q252H)-phosphorylated	84	CDKL1	89	ERBB4	100
ABL1(T315I)-nonphosphorylated	94	CDKL2	100	ERK1	98
ABL1(T315I)-phosphorylated	84	CDKL3	100	ERK2	100
ABL1(Y253F)-phosphorylated	72	CDKL5	72	ERK3	97
ABL1-nonphosphorylated	75	CHEK1	100	ERK4	98
ABL1-phosphorylated	70	CHEK2	100	ERK5	100
ABL2	100	CIT	100	ERK8	100
ACVR1	100	CLK1	100	ERN1	84
ACVR1B	97	CLK2	74	FAK	100
ACVR2A	100	CLK3	100	FER	95
ACVR2B	93	CLK4	100	FES	100
ACVRL1	100	CSF1R	99	FGFR1	100
ADCK3	100	CSF1R-autoinhibited	98	FGFR2	100
ADCK4	100	CSK	100	FGFR3	100
AKT1	100	CSNK1A1	79	FGFR3 (G697C)	96
AKT2	100	CSNK1A1L	100	FGFR4	100
AKT3	100	CSNK1D	99	FGR	100
ALK	97	CSNK1E	99	FLT1	100
ALK(C1156Y)	79	CSNK1G1	100	FLT3	93
ALK(L1196M)	75	CSNK1G2	98	FLT3(D835H)	100
AMPK-alpha1	100	CSNK1G3	100	FLT3(D835V)	73
AMPK-alpha2	83	CSNK2A1	79	FLT3(D835Y)	100
ANKK1	87	CSNK2A2	86	FLT3(ITD)	100
ARK5	100	CTK	79	FLT3(ITD,D835V)	84
ASK1	99	DAPK1	100	FLT3(ITD,F691L)	71
ASK2	95	DAPK2	96	FLT3(K663Q)	100
AURKA	83	DAPK3	95	FLT3(N841I)	94
AURKB	86	DCAMKL1	95	FLT3(R834Q)	91
AURKC	100	DCAMKL2	93	FLT3-autoinhibited	77
AXL	99	DCAMKL3	100	FLT4	79
BIKE	87	DDR1	100	FRK	100
BLK	95	DDR2	80	FYN	100
BMPR1A	84	DLK	98	GAK	100
BMPR1B	90	DMPK	94	GCN2(Kin.Dom.2,S808G)	94
BMPR2	93	DMPK2	96	GRK1	92
BMX	100	DRAK1	100	GRK2	94
BRAF	88	DRAK2	100	GRK3	74
BRAF(V600E)	94	DYRK1A	79	GRK4	95
BRK	100	DYRK1B	66	GRK7	100
BRSK1	100	DYRK2	84	GSK3A	91
BRSK2	100	EGFR	100	GSK3B	94
BTK	66	EGFR(E746-A750del)	100	HASPIN	80
BUB1	89	EGFR(G719C)	98	HCK	100
CAMK1	92	EGFR(G719S)	100	HIPK1	94
CAMK1B	100	EGFR(L747-E749del, A750P)	100	HIPK2	77
CAMK1D	96	EGFR(L747-S752del, P753S)	100	HIPK3	90
CAMK1G	100	EGFR(L747-T751del,Sins)	100	HIPK4	100
CAMK2A	89	EGFR(L858R)	100	HPK1	80
CAMK2B	96	EGFR(L858R,T790M)	98	HUNK	86
CAMK2D	100	EGFR(L861Q)	100	ICK	93
CAMK2G	100	EGFR(S752-I759del)	100	IGF1R	97
CAMK4	87	EGFR(T790M)	89	IKK-alpha	73
CAMKK1	100	EIF2AK1	98	IKK-beta	87
CAMKK2	100	EPHA1	100	IKK-epsilon	76
CASK	87	EPHA2	95	INSR	91
CDC2L1	96	EPHA3	95	INSRR	99
CDC2L2	93	EPHA4	82	IRAK1	96
CDC2L5	84	EPHA5	100	IRAK3	100

DiscoveryX Gene Symbol	Percent control	DiscoveryX Gene Symbol	Percent control	DiscoveryX Gene Symbol	Percent control
IRAK4	86	MST1	96	PIM2	100
ITK	100	MST1R	100	PIM3	88
JAK1(JH1domain-catalytic)	100	MST2	99	PIP5K1A	94
JAK1(JH2domain-pseudokinase)	59	MST3	92	PIP5K1C	95
JAK2(JH1domain-catalytic)	83	MST4	91	PIP5K2B	100
JAK3(JH1domain-catalytic)	81	MTOR	34	PIP5K2C	84
JNK1	91	MUSK	100	PKAC-alpha	100
JNK2	85	MYLK	82	PKAC-beta	100
JNK3	86	MYLK2	99	PKMYT1	100
KIT	96	MYLK4	93	PKN1	100
KIT(A829P)	34	MYO3A	100	PKN2	100
KIT(D816H)	68	MYO3B	100	PKNB(M.tuberculosis)	81
KIT(D816V)	100	NDR1	93	PLK1	93
KIT(L576P)	89	NDR2	89	PLK2	78
KIT(V559D)	98	NEK1	100	PLK3	77
KIT(V559D,T670I)	98	NEK10	80	PLK4	83
KIT(V559D,V654A)	100	NEK11	85	PRKCD	100
KIT-autoinhibited	83	NEK2	100	PRKCE	92
LATS1	99	NEK3	79	PRKCH	100
LATS2	74	NEK4	95	PRKCI	88
LCK	99	NEK5	98	PRKCQ	100
LIMK1	100	NEK6	100	PRKD1	100
LIMK2	97	NEK7	100	PRKD2	96
LKB1	92	NEK9	100	PRKD3	100
LOK	100	NIK	98	PRKG1	100
LRRK2	89	NIM1	79	PRKG2	84
LRRK2(G2019S)	96	NLK	100	PRKR	100
LTK	98	OSR1	77	PRKX	100
LYN	99	p38-alpha	91	PRP4	97
LZK	95	p38-beta	87	PYK2	100
MAK	100	p38-delta	100	QSK	86
MAP3K1	94	p38-gamma	92	RAF1	100
MAP3K15	69	PAK1	99	RET	100
MAP3K2	91	PAK2	97	RET(M918T)	98
MAP3K3	100	PAK3	100	RET(V804L)	100
MAP3K4	95	PAK4	100	RET(V804M)	100
MAP4K2	85	PAK6	99	RIOK1	91
MAP4K3	100	PAK7	99	RIOK2	18
MAP4K4	94	PCTK1	67	RIOK3	98
MAP4K5	96	PCTK2	100	RIPK1	97
MAPKAPK2	100	PCTK3	95	RIPK2	100
MAPKAPK5	83	PDGFRA	81	RIPK4	84
MARK1	100	PDGFRB	97	RIPK5	74
MARK2	99	PDPK1	100	ROCK1	86
MARK3	100	PFCDPK1(P.falciparum)	89	ROCK2	85
MARK4	100	PFPK5(P.falciparum)	95	ROS1	96
MAST1	98	PFTAIRE2	100	RPS6KA4(Kin.Dom.1-N-terminal)	100
MEK1	86	PFTK1	100	RPS6KA4(Kin.Dom.2-C-terminal)	88
MEK2	83	PHKG1	100	RPS6KA5(Kin.Dom.1-N-terminal)	100
MEK3	92	PHKG2	97	RPS6KA5(Kin.Dom.2-C-terminal)	95
MEK4	96	PIK3C2B	64	RSK1(Kin.Dom.1-N-terminal)	95
MEK5	75	PIK3C2G	79	RSK1(Kin.Dom.2-C-terminal)	97
MEK6	100	PIK3CA	45	RSK2(Kin.Dom.1-N-terminal)	93
MELK	93	PIK3CA(C420R)	62	RSK2(Kin.Dom.2-C-terminal)	100
MERTK	99	PIK3CA(E542K)	47	RSK3(Kin.Dom.1-N-terminal)	100
MET	98	PIK3CA(E545A)	44	RSK3(Kin.Dom.2-C-terminal)	100
MET(M1250T)	100	PIK3CA(E545K)	52	RSK4(Kin.Dom.1-N-terminal)	85
MET(Y1235D)	100	PIK3CA(H1047L)	20	RSK4(Kin.Dom.2-C-terminal)	98
MINK	82	PIK3CA(H1047Y)	40	S6K1	94
MKK7	80	PIK3CA(I800L)	21	SBK1	81
MKNK1	72	PIK3CA(M1043I)	39	SGK	91
MKNK2	74	PIK3CA(Q546K)	51	SgK110	100
MLCK	100	PIK3CB	9.8	SGK2	86
MLK1	93	PIK3CD	43	SGK3	80
MLK2	100	PIK3CG	53	SIK	100
MLK3	100	PIK4CB	71	SIK2	100
MRCKA	100	PIKFYVE	0	SLK	98
MRCKB	99	PIM1	83	SNARK	90

DiscoverX Gene Symbol	Percent control	DiscoverX Gene Symbol	Percent control	DiscoverX Gene Symbol	Percent control
SNRK	87	TGFBR2	99	ULK1	81
SRC	100	TIE1	100	ULK2	91
SRMS	89	TIE2	99	ULK3	98
SRPK1	89	TLK1	96	VEGFR2	93
SRPK2	100	TLK2	100	VPS34	65
SRPK3	100	TNIK	100	VRK2	79
STK16	97	TNK1	89	WEE1	100
STK33	86	TNK2	100	WEE2	100
STK35	99	TNNI3K	93	WNK1	71
STK36	96	TRKA	94	WNK2	77
STK39	100	TRKB	97	WNK3	66
SYK	96	TRKC	90	WNK4	40
TAK1	95	TRPM6	100	YANK1	96
TAOK1	94	TSSK1B	100	YANK2	100
TAOK2	83	TSSK3	84	YANK3	94
TAOK3	93	TTK	90	YES	100
TBK1	98	TXK	99	YSK1	100
TEC	100	TYK2(JH1domain-catalytic)	89	YSK4	82
TESK1	95	TYK2(JH2domain-pseudokinase)	75	ZAK	91
TGFBR1	100	TYRO3	73	ZAP70	92

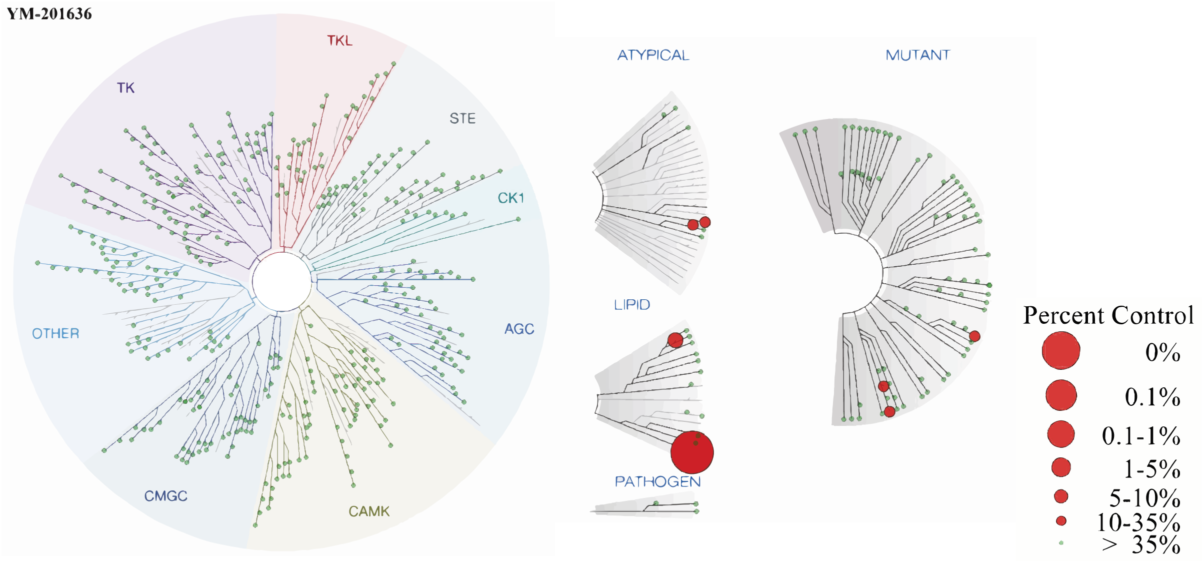

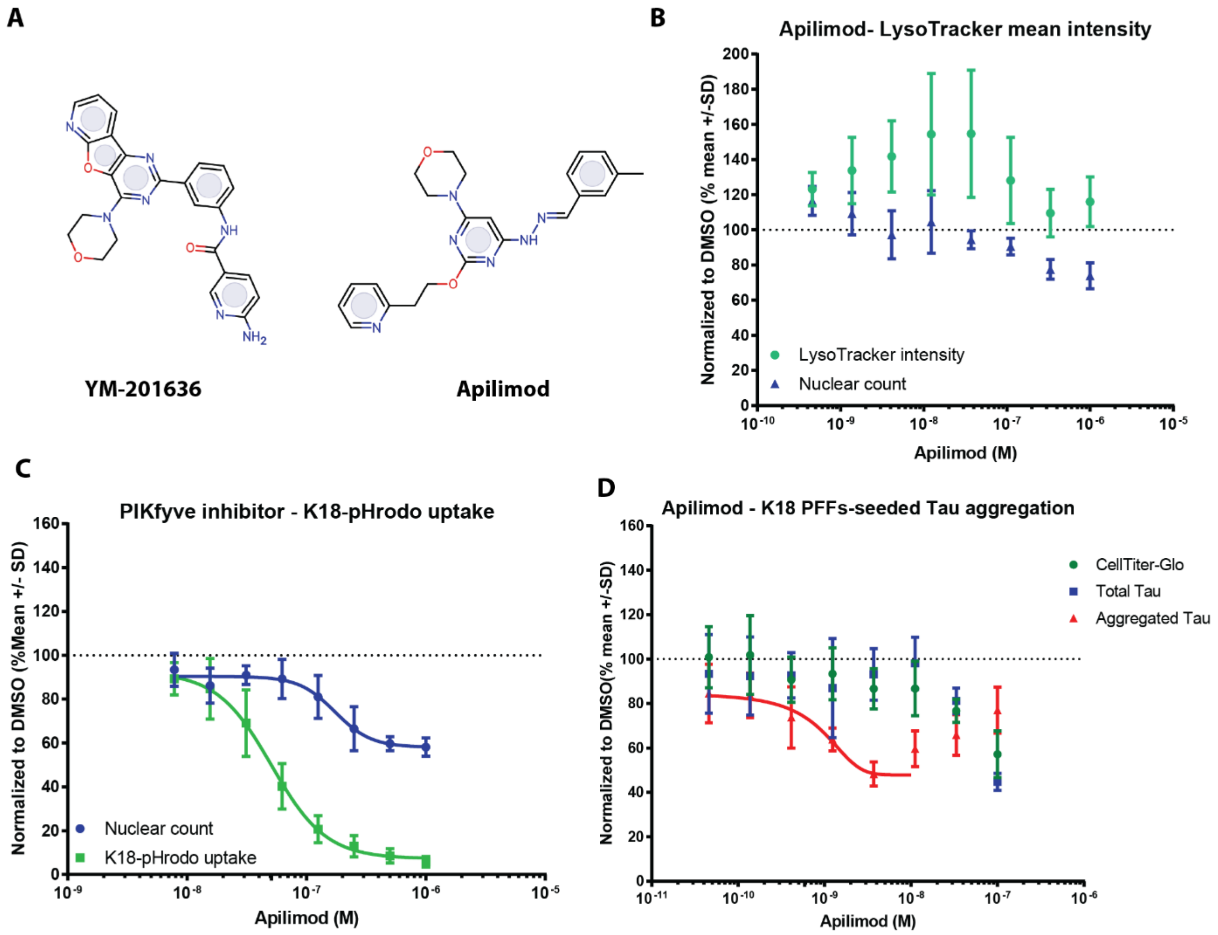

Supporting figure 7 – (A) Chemical structure of YM-201636 and Apilimod. (B) Image quantification of the intensity of Lysotracker positive vesicles of primary hippocampal cultures treated with different concentrations of Apilimod. (C) Image quantification of K18-pHrodo signal in primary hippocampal cultures treated with the PIKfyve inhibitor Apilimod in a dose-response (n=3). (D) CellTiter-Glo, total and aggregated Tau measured in primary hippocampal cultures treated with Apilimod and 50nM K18 PFFs to induce Tau aggregation in neurons over-expressing Tau:P301L (n=3).

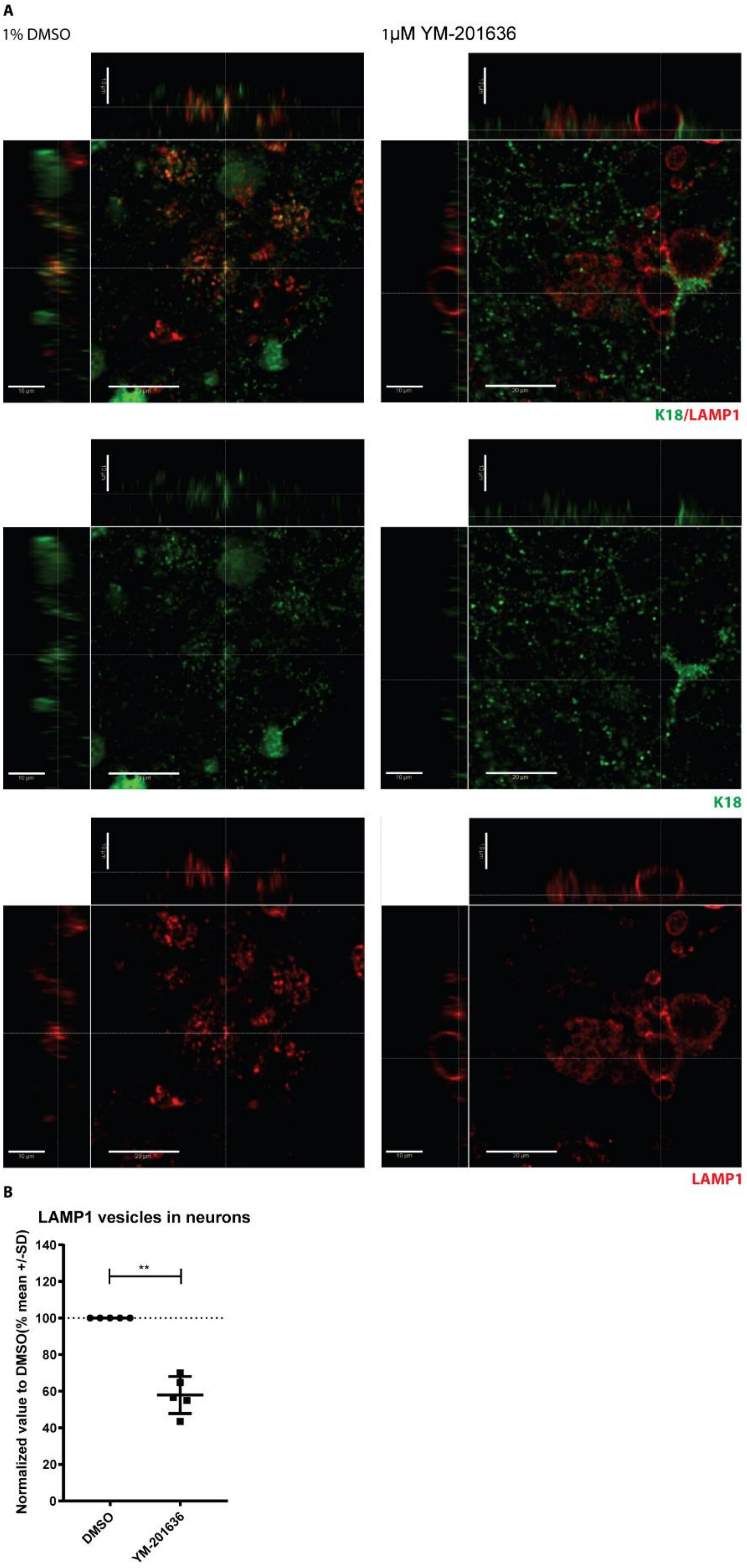

Supporting figure 8 – (A)Representative images of XYZ projections of primary hippocampal neurons treated with either 1µM YM-201636 or 1% DMSO before incubation with 250nM K18-AF488 PFFs. Primary hippocampal neurons were incubated with labelled Tau seeds for 16 hours before being fixed and stained for LAMP1 and MAP2 (not shown). Samples were imaged using a 40x water objective and XYZ projection was performed using the Harmony software (PerkinElmer) on 40 image planes (scale bar on XY view: 20µm, scale bar on XYZ view: 10µm). (B) Quantification of LAMP1 vesicles in neurons treated with 1% DMSO or 1 µM YM-201636 (n=5).

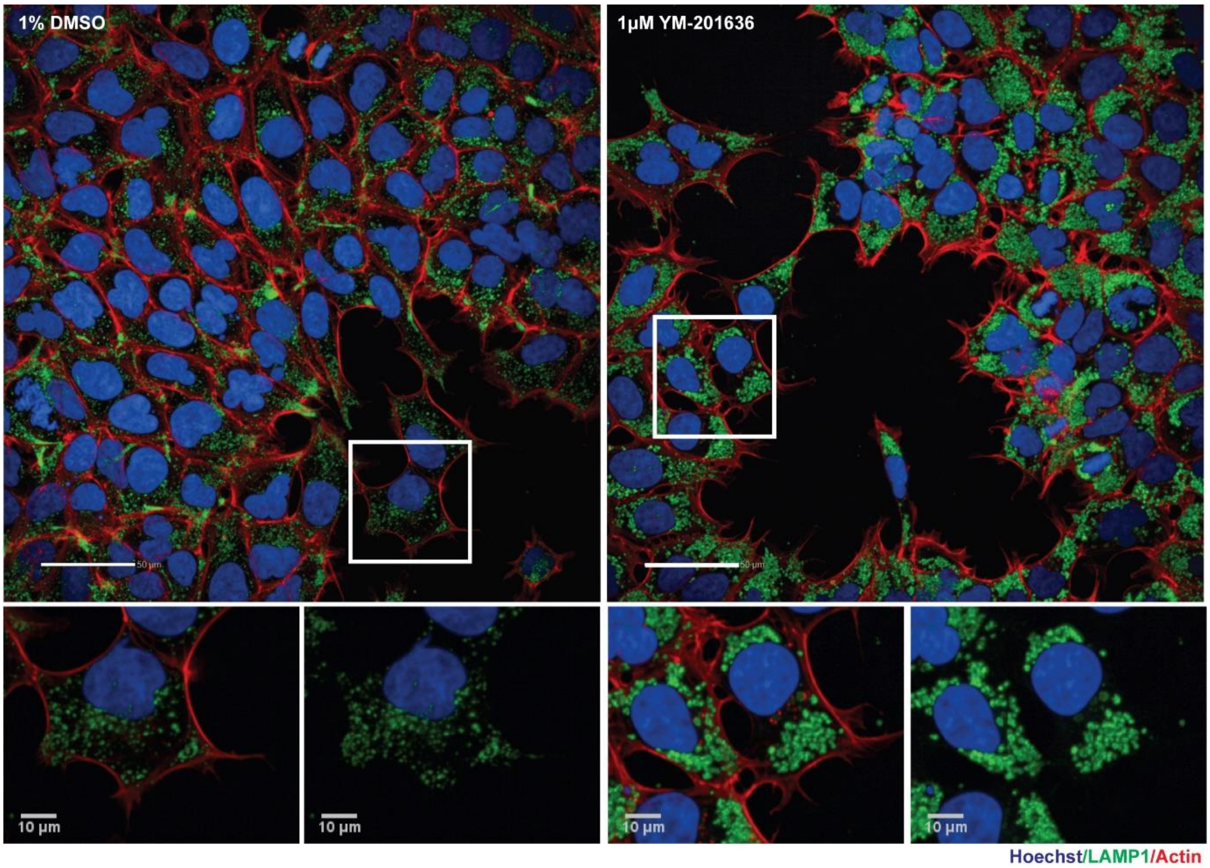

Supporting figure 9 - Representative images of HEK-QBi cells treated with either 1% DMSO or 1µM YM-201636 compound for 30 minutes, fixed and immunostained for LAMP1 and for actin using phalloidin (scale bar of main figures: 50µm).
